# Natural TCRs targeting KRAS^G12V^ display fine specificity and sensitivity to human solid tumors

**DOI:** 10.1172/JCI175790

**Published:** 2024-09-17

**Authors:** Adham S. Bear, Rebecca B. Nadler, Mark H. O’Hara, Kelsey L. Stanton, Chong Xu, Robert J. Saporito, Andrew J. Rech, Miren L. Baroja, Tatiana Blanchard, Maxwell H. Elliott, Michael J. Ford, Richard Jones, Shivang Patel, Andrea Brennan, Zachary O’Neil, Daniel J. Powell, Robert H. Vonderheide, Gerald P. Linette, Beatriz M. Carreno

**Affiliations:** 1 Division of Hematology-Oncology, Department of Medicine, Perelman School of Medicine,; 2 The College of Arts and Sciences,; 3 Abramson Cancer Center, and; 4 Center for Cellular Immunotherapies, Perelman School of Medicine, University of Pennsylvania, Philadelphia, Pennsylvania, USA.; 5 MSBioworks, Ann Arbor, Michigan, USA.; 6 Department of Pathology and Laboratory Medicine and; 7 Parker Institute for Cancer Immunotherapy, Perelman School of Medicine, University of Pennsylvania, Philadelphia, Pennsylvania, USA.

**Keywords:** Immunology, Oncology, Antigen presentation, Cancer immunotherapy, T cell receptor

## Abstract

**BACKGROUND:**

Neoantigens derived from KRAS^MUT^ have been described, but the fine antigen specificity of T cell responses directed against these epitopes is poorly understood. Here, we explore KRAS^MUT^ immunogenicity and the properties of 4 T cell receptors (TCRs) specific for KRAS^G12V^ restricted to the HLA-A3 superfamily of class I alleles.

**METHODS:**

A phase 1 clinical vaccine trial targeting KRAS^MUT^ was conducted. TCRs targeting KRAS^G12V^ restricted to HLA-A*03:01 or HLA-A*11:01 were isolated from vaccinated patients or healthy individuals. A comprehensive analysis of TCR antigen specificity, affinity, crossreactivity, and CD8 coreceptor dependence was performed. TCR lytic activity was evaluated, and target antigen density was determined by quantitative immunopeptidomics.

**RESULTS:**

Vaccination against KRAS^MUT^ resulted in the priming of CD8^+^ and CD4^+^ T cell responses. KRAS^G12V^ -specific natural (not affinity enhanced) TCRs exhibited exquisite specificity to mutated protein with no discernible reactivity against KRAS^WT^. TCR-recognition motifs were determined and used to identify and exclude crossreactivity to noncognate peptides derived from the human proteome. Both HLA-A*03:01 and HLA-A*11:01–restricted TCR-redirected CD8^+^ T cells exhibited potent lytic activity against KRAS^G12V^ cancers, while only HLA-A*11:01–restricted TCR-T CD4^+^ T cells exhibited antitumor effector functions consistent with partial coreceptor dependence. All KRAS^G12V^-specific TCRs displayed high sensitivity for antigen as demonstrated by their ability to eliminate tumor cell lines expressing low levels of peptide/HLA (4.4 to 242) complexes per cell.

**CONCLUSION:**

This study identifies KRAS^G12V^-specific TCRs with high therapeutic potential for the development of TCR-T cell therapies.

**TRIAL REGISTRATION:**

ClinicalTrials.gov NCT03592888.

**FUNDING:**

AACR SU2C/Lustgarten Foundation, Parker Institute for Cancer Immunotherapy, and NIH.

## Abstract 2

This study reports on a dendritic cell-based vaccine against KRAS peptides in PAAD patients and describes the functional characteristics of four KRAS G12V-specific TCRs restricted to the HLA-A3 superfamily of class I alleles with potential for development of adoptive T cell therapies.

## Introduction

T cell recognition of cancer antigens represents the end effector mechanism of successful cancer immunotherapy (1). Advances in the areas of T cell biology, gene engineering, and antigen identification have nurtured strategies to redirect T cell antigen specificity against cancer cells. Indeed, CAR-T cell therapy for the treatment of hematological malignancies is now widely available (2). Adoptive cell therapy strategies utilizing T cells redirected with tumor-specific T cell receptors (TCRs) (TCR-T) have demonstrated promising clinical results in subsets of cancer patients (3–5); however, fundamental challenges exist, including antigen identification.

Neoantigens arising from recurrent activating mutations in oncogenic driver genes are attractive immunotherapeutic targets due to limited clonal heterogeneity and treatment generalizability across patients and tumor types (6). To date, shared neoantigens of mutant *TP53*, *PIKC3A*, and *KRAS* among others have been described (7). KRAS mutations are observed in up to 20% of all human cancers and drive tumorigenesis in the 3 most lethal cancers in the United States, including adenocarcinomas of the pancreas (PAAD: 80%–90%), colon (COAD: 40%–50%), and lung (LUAD: 30%–40%) (8). The vast majority of KRAS mutations in these tumors occur at the codon 12 position (9), leading to hyperactivation of MAPK and PI3K-AKT downstream effector signaling pathways (10). Among these tumor types, amino acid substitutions at codon position 12 most often involve glycine (G) to aspartic acid (D), valine (V), cysteine (C), or arginine (R) transitions. While G12C and G12R mutations are preferentially observed among LUAD and PAAD tumors, respectively, G12D and G12V mutations remain highly prevalent across the 3 tumor types. Clinical case reports suggest mutant KRAS (KRAS^MUT^) may be amenable to targeting by TCR-based therapies in select patients with KRAS^G12D^ tumors who are HLA-C*08:02^+^ (11, 12). Immunopeptidomic studies performed by our group and others highlight the HLA-A3 superfamily of class I alleles (A*03:01, A*11:01, A*31:01, A*33:01, and others), which share overlapping peptide repertoires (13, 14), as capable of processing and presenting nomaner and decamer epitopes of KRAS^WT^ and KRAS^MUT^ spanning amino acid residues 8–16 (VVGAXGVGK) and 7–16 (VVVGAXGVGK), respectively, with X signifying the amino acid at codon position 12 (15–17).

The CD8 coreceptor functions to enhance TCR avidity through stabilization of the TCR:peptide/HLA (pHLA) immune synapse via binding to the α3 domain of HLA-I molecules (18). An inherent limitation of TCR-T therapy using HLA-I–restricted TCRs is an inability to leverage CD4^+^ T cell immunity. CD4^+^ T cells may be directly cytotoxic and promote antigen-specific help during several phases of the immune response that improves the in vivo persistence and antitumor activity of tumor-specific CD8^+^ T cells (19). Transgenic expression of CD8αβ may enhance the antitumor activity of CD4^+^ T cells redirected with HLA-I–restricted TCRs, but such a strategy requires further engineering of T cells. Select TCRs of high pHLA avidity function independently of CD8 coreceptor interactions, allowing transgenic TCR expression on CD4^+^ T cells to recruit their effector functions (20, 21).

Here, we report a phase 1 clinical trial of autologous mature dendritic cell (mDC) vaccination targeting KRAS^MUT^ (mDC3/8-KRAS). Vaccination resulted in the priming of T cell immunity against KRAS^MUT^ in select subjects, including CD8^+^ T cell immunity against KRAS^G12V^ in an HLA-A*11:01^+^ patient. We further explored KRAS^G12V^ restricted to HLA-A*03:01 and HLA-A*11:01 as immunological targets using a panel of 4 TCRs derived from this vaccinated cancer patient and healthy donors. TCRs were highly specific to KRAS^G12V^ without crossreactivity to predicted peptides encoded in the human proteome and displayed various degrees of CD8 coreceptor dependence. HLA-A*11:01–restricted TCR-engineered CD8^+^ and CD4^+^ T cells exhibited lytic activity against KRAS^G12V+^ tumor cell lines with low-abundance neoantigen expression. These results validate G12V/A*03:01 and G12V/A*11:01 as shared neoantigen targets, which underlies the development of adoptive TCR-T cell therapies for the treatment of KRAS^MUT^ cancers.

## Results

### Vaccination primes KRAS^MUT^-specific T cell responses in pancreatic cancer patients.

**Figure 1:**
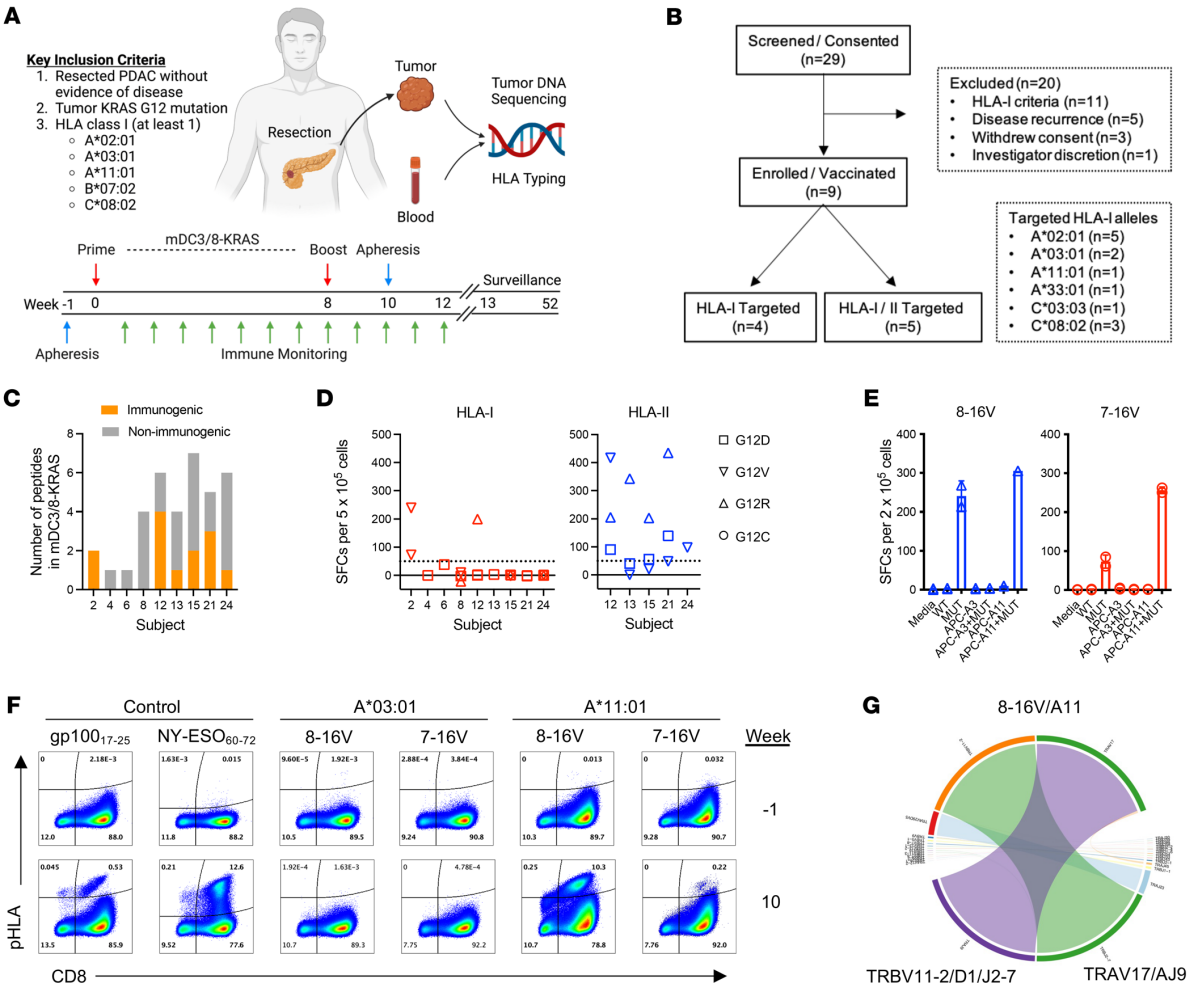
mDC3/8-KRAS vaccination primes KRAS^MUT^-specific T cell immunity in PAAD patients. (**A**) Trial design. (**B**) Consolidated standards of reporting trials diagram. (**C**) Number of vaccine KRAS^MUT^ neoantigens per patient that induced IFN-γ^+^ T cells in ex vivo–expanded PBMCs collected after vaccine priming. (**D**) Normalized IFN-γ^+^ ELISpot counts for vaccine KRAS^MUT^ neoantigens after priming detected in ex vivo–expanded PBMCs. Spot counts of the nonstimulated controls were subtracted. Responses to short peptides (HLA-I) are indicated in red, and responses to long peptides (HLA-II) are indicated in blue. Symbol shape indicates specific KRAS^MUT^ as per legend. (**E**) Assessment of subject no. 2 HLA-I–restricted T cell responses against 8–16V (blue) and 7–16V (red) peptides by IFN-γ ELISpot assay following ex vivo expansion of week 2 postvaccine PBMCs. Free peptide supplemented to media bound by HLA-I expressed on donor white blood cells (HLA-A*11:01 and -A*03:01) and presented to responding T cells. Monoallelic K562 cells expressing HLA-A*03:01 (APC-A3) or HLA-A*11:01 (APC-A11) were used to identify HLA-I restriction. WT indicates WT KRAS peptide. *MUT* indicates mutant KRAS peptide. (**F**) pHLA multimer analysis to assess CD8^+^ T cell response against 8–16V/A*11:01 and 7–16V/A*11:01 following in vitro expansion of pre- (week –1) and postvaccine (week 10) CD8^+^ T cells. Successful priming of CD8^+^ T cell responses to gp100_17–25_/A*03:01 and NY-ESO_60–72_/B*07:02 served as positive vaccination controls. (**G**) Circos plot analysis following TCR-αβ RNA sequencing of FACS-sorted CD8^+^/multimer^+^ (8–16V/A*11:01) cells. Statistical differences between groups calculated using Students’ unpaired *t* test.

We conducted an investigator-initiated, phase 1 clinical trial to study the immunogenicity of KRAS^MUT^ in PAAD patients (ClinicalTrials.gov NCT03592888) utilizing a previously described autologous, mDC-based (mDC3/8) platform (Figure 1A) (22). Key enrollment criteria included (a) a history of resected PAAD without radiographic or biochemical evidence of disease, (b) the presence of a tumor KRAS^G12^ mutation determined by tumor DNA sequencing, and (c) patient expression of at least 1 HLA-I allele corresponding to previously reported KRAS^MUT^ neoantigens (15–17). From July 19, 2018, to April 17, 2024, we enrolled 29 subjects of which 9 subjects received vaccination (Figure 1B). All vaccinated subjects had clinical characteristics typical of patients with resected PAAD (Supplemental Table 1; supplemental material available online with this article; https://doi.org/10.1172/JCI175790DS1). All 9 subjects were vaccinated against 1 or more distinct short (nonamer or decamer) KRAS^MUT^ peptides targeting patient-specific HLA-I alleles, and 5 of the 9 patients were also vaccinated against 1 or more distinct long KRAS^MUT^ peptides in order to, presumably, target HLA-II alleles (Figure 1B and Supplemental Table 2). The number of peptides administered to each patient ranged from 1 to 7 peptides representing KRAS^MUT^ variant present in the patient’s tumor as well as others (Supplemental Table 3).

All vaccinated subjects received a total of 2 vaccine doses (prime and boost) i.v. Two subjects were vaccinated below the prespecified prime or boost dose levels of 10–30 × 10^6^ and 7–15 × 10^6^ DCs/peptide, respectively (Supplemental Figure 1A). Effective DC maturation using CD40L/IFN-γ, poly I:C, and R848 was confirmed in all subjects via the induction of IL-12p70 production (Supplemental Figure 1B). Vaccination was safe and well tolerated (primary end points) with no subjects experiencing grade 3 or higher adverse events (AEs). The most common AEs observed following vaccination included chills, fatigue, and headache (Supplemental Figure 1C).

KRAS^MUT^-specific T cell responses primed by vaccination (secondary endpoint) were assessed by IFN-γ ELISpot assay following in vitro T cell expansion. Six out of 9 (67%) subjects generated KRAS^MUT^-specific T cell responses following vaccination (Figure 1C). In a subset of subjects, T cell responses were directed at more than one KRAS^MUT^ peptide. Two (subjects nos. 2 and 12) out of 9 subjects generated T cell responses to HLA-I–restricted short peptides, whereas the inclusion of long KRAS^MUT^ peptides in the vaccine formulation enhanced the immune response rate with 5/5 subjects exhibiting measurable immunity to at least 1 long peptide (Figure 1D). Whether these long peptides elicited CD4 and/or CD8 T cell responses is still being characterized. At a median time to follow-up of 25.3 months, 5 subjects were alive without evidence of tumor recurrence, while 4 subjects had experienced tumor recurrence and died due to disease progression (Supplemental Figure 1D).

Subject 2 was vaccinated against nonamer 8–16V and decamer 7–16V KRAS^MUT^ peptides targeting patient HLA-I alleles A*03:01 and A*11:01 along with control peptides gp100 (gp_17–25_) restricted to HLA-A*03:01 and NY-ESO-1 (NY_60–72_) restricted to HLA-B*07:02. Analysis of ex vivo–expanded PBMC samples collected at week 2 after vaccination demonstrated a positive immune response against both 8–16V and 7–16V with no reactivity to KRAS^WT^ peptides (Figure 1E). To identify the HLA-I–restricting allele of this response, K562 cells (HLA-I negative) were engineered to express a single-chain dimer construct encoding β_2_-microglobulin linked to either HLA-A*03:01 (K562^A*03:01^) or HLA-A*11:01 (K562^A*11:01^) heavy chain (Supplemental Figure 1E) (23). IFN-γ production by ex vivo–expanded PBMC samples was detected in the presence of 8–16V or 7–16V peptide-pulsed K562^A*11:01^ but not K562^A*03:01^ cells, indicating this response to be restricted by HLA-A*11:01 and not HLA-A*03:01. pHLA multimer analysis of ex vivo–expanded CD8^+^ T cells collected before (week –1) and after vaccination (week 10) confirmed de novo priming of a 8–16V/A*11:01–specific CD8^+^ T cell response and a weaker priming of a 7–16V/A*11:01 response (Figure 1F). No HLA-A*03:01–restricted T cell response against KRAS^G12V^ was observed despite successful vaccine-induced priming of CD8^+^ T cell responses against gp_17–25_/A*03:01. In subject 24, vaccination against 8–16V and 7–16V KRAS^MUT^ peptides targeted to HLA-A*03:01 did not elicit an immune response; however, this patient did not exhibit a response against control gp_17–25_ peptide, suggesting impaired CD8^+^ T cell immunity (Supplemental Figure 1F). Subject 12 demonstrated a positive CD8^+^ T cell response against KRAS^G12R^ peptide 3–12R by IFN-γ ELISpot and pHLA multimer assays (Supplemental Figure 1, G and H), a candidate KRAS^MUT^ neoantigen with predicted high-binding affinity to HLA-A*33:01 (affinity = 10.5 nM, NetMHC4.0). Notably, no HLA-A*02:01– or HLA-C*08:02–restricted KRAS^MUT^ peptide-specific immune responses were observed in vaccinated subjects exhibiting these HLA-I alleles (Supplemental Table 3).

### Identification of KRAS^G12V^-specific TCRs in healthy donors and vaccinated patients.

In prior work, we utilized a multiomic approach to identify HLA-I–restricted neoantigens derived from KRAS^MUT^ (15). We performed biochemical studies to measure pHLA-binding affinity and complex stability, both parameters that correlate with peptide immunogenicity. These studies highlight epitopes of KRAS^G12V^ (8–16V and 7–16V) restricted to HLA-A*03:01 and HLA-A*11:01 as exhibiting optimal immunogenic properties relative to other KRAS^MUT^ epitopes (Supplemental Figure 1I). In select healthy donors, in vitro priming and T cell expansion assays yielded CD8^+^ T cell responses against the 7–16V epitope restricted to HLA-A*03:01 and HLA-A*11:01, but not the 8–16V peptide (Supplemental Figure 1J). From these donors, we isolated TCR-αβ pairs specific for 7–16V/A*03:01 (designated as A3V) and 7–16V/A*11:01 (designated as A11Va, A11Vb) (Table 1). Additionally, a TCR-αβ pair specific for 8–16V/A*11:01 (designated as A11Vc) was isolated from an oligoclonal population identified in vaccine subject 2 (Figure 1G and Table 1). Both A3V and A11Va have been previously reported (15), while A11Vb and A11Vc are first introduced here.

### TCRs are highly specific for KRAS^G12V^ and recognize distinct peptide-binding motifs.

**Figure 2:**
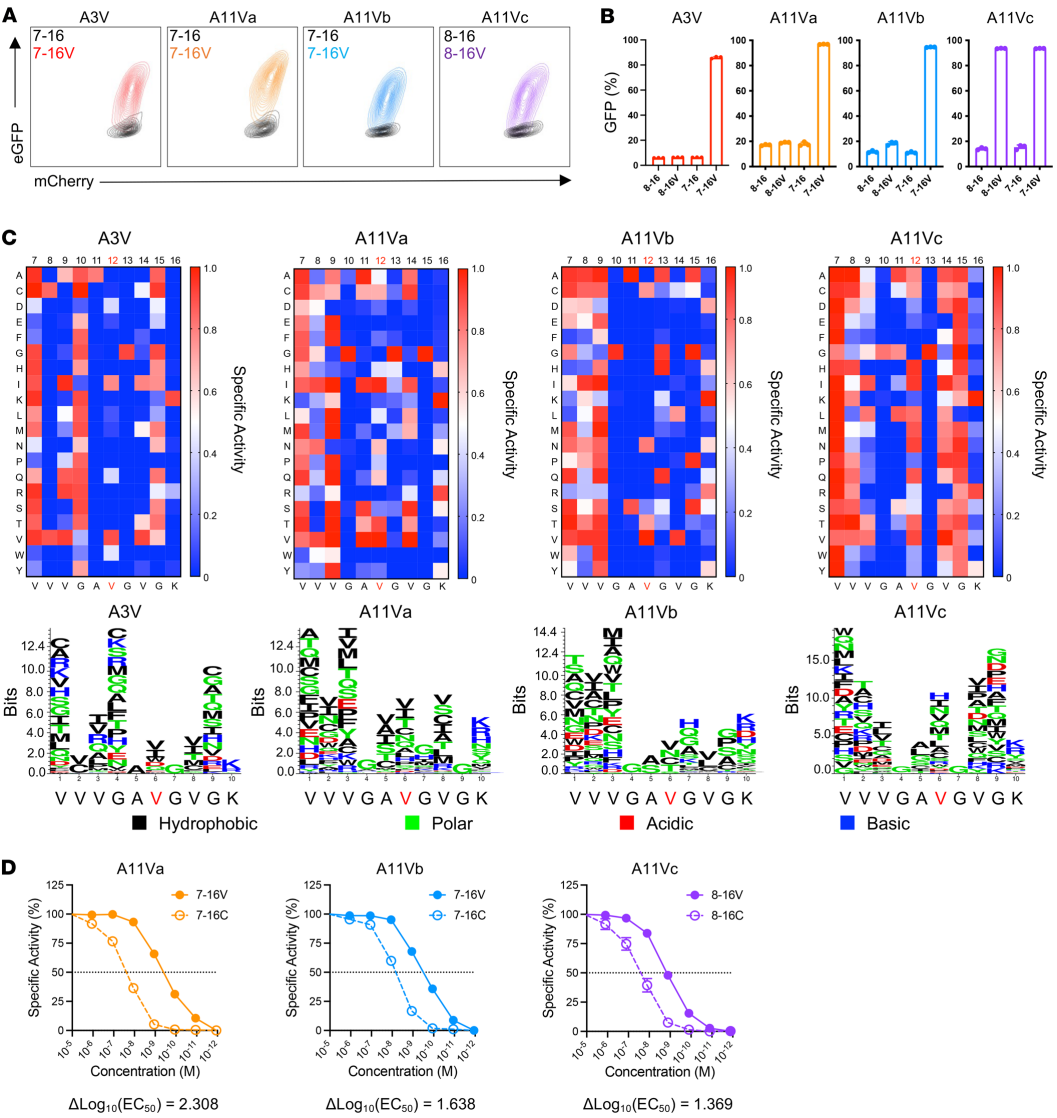
TCRs are specific for KRAS^G12V^ and exhibit distinct peptide-binding motifs with crossreactivity to KRAS^G12C^. (**A**) FACS profiles of TCR-engineered J^ASP90_C^D8^+^ cells following 16 hours of coculture with HLA-I –matched K562 cells pulsed with KRAS^WT^ (black) or cognate KRAS^G12V^ (colored) peptide. (**B**) Bar graphs representing NFAT activation (specific activity, %) of J^ASP90_C^D8^+^ cells following 16 hours of coculture with HLA-I–matched K562 cells pulsed with 9-mer and 10-mer KRAS^WT^ or KRAS^G12V^ peptides. (**C**) Peptide-binding motifs determined by X-scan analysis of TCR A3V, A11Va, A11Vb, and A11Vc depicted as heatmaps (top) and Seq2Logo plots (bottom) using J^ASP90_C^D8^+^ reporter cells cocultured with HLA-I–matched K562 cells pulsed with positional peptide scanning library peptides. Heatmaps: specific activity = (GFP_Exp_ - GFP_Min_) / (GFP_Max_ – GFP_Min_); GFP_Min_ = unstimulated, GFP_Max_ = PMA-I. Seq2Logo plots: height of amino acid at each position corresponds to EGFP expression relative to unstimulated and PMA-I conditions. (**D**) Cell-reporter assay using TCR-engineered J^ASP90_C^D8^+^ cocultured with K562^A*11:01^ cells pulsed with titrated levels of cognate G12V versus G12C peptides. Differences in TCR functional avidities for each peptide are displayed as Δlog_10_(EC_50_) values. Data are representative of 2 or more experiments.

We utilized J^ASP90_C^D8^+^ reporter cells, which comprise TCR-αβ^null^ Jurkat E6.1 cells engineered to express the CD8αβ coreceptor and a Uni-Vect reporter construct consisting of an nuclear factor of activated T cells–inducible (NFAT-inducible) EGFP reporter to readout TCR signaling (23). J^ASP90_C^D8^+^ cells were further engineered to express KRAS^MUT^ TCR constructs via lentiviral transduction and positively sorted to purity based on CD3 expression (Supplemental Figure 2A). TCR-engineered J^ASP90_C^D8^+^ cells were cocultured with HLA-I–matched K562 cells pulsed with either KRAS^WT^ or KRAS^G12V^ synthetic peptides and assessed for TCR activation (EGFP expression) 16 hours later. All TCRs demonstrated specific reactivity to cognate KRAS^G12V^ peptides without crossreactivity to KRAS^WT^ (Figure 2A). A3V, A11Va, and A11Vb were exclusively reactive against 7–16V, whereas A11Vc exhibited reactivity to 8–16V and 7–16V peptides (Figure 2B).

To define peptide residues critical for TCR engagement (recognition motif), we initially employed an Ala/Gly peptide library (Supplemental Data Set 1) for presentation by HLA-I–matched K562 cells and cocultured with TCR-engineered J^ASP90_C^D8^+^ cells. Ala/Gly scanning assays identified both anchor (peptide position 2 [P2]) and nonanchor (P4–P8) residues within the 7–16V peptide critical for the activation of each TCR (Supplemental Figure 2B). TCR recognition motif and crossreactivity characterization were explored further by employing a positional peptide library, X-scan (Figure 2C) (24, 25). The X-scan library consisted of 190 synthetic peptides in which each amino acid residue in the 7–16V peptide sequence was substituted by all 19 remaining l-amino acids (Supplemental Data Set 2). As expected, limited amino acid substitutions were tolerated at residues P2/3 and P10, corresponding to N- and C-terminal anchor positions, respectively. Notably, A11Vc functioned independently of all amino acid substitutions at P1 of the 10-mer peptide while A11Va and A11Vb recognition was affected by select P1 amino acid substitutions. For all TCRs, limited substitutions were tolerated for valine at P6 corresponding to the codon 12 mutant position. A11Va-c demonstrated crossreactivity to KRAS^G12C^ (7–16C), which is the most prevalent KRAS mutation observed in human LUAD. We compared the functional avidities of A11Va-c against cognate KRAS^G12V^ versus KRAS^G12C^ peptides using TCR-engineered J^ASP90_C^D8^+^ cells cocultured with K562^A*11:01^ cells pulsed with titrated peptide concentrations. A11Va-c exhibited approximately 10- to 100-fold greater functional avidity against cognate KRAS^G12V^ as compared with KRAS^G12C^ peptides (Figure 2D).

### Assessment of TCR crossreactivity to the human proteome.

**Figure 3:**
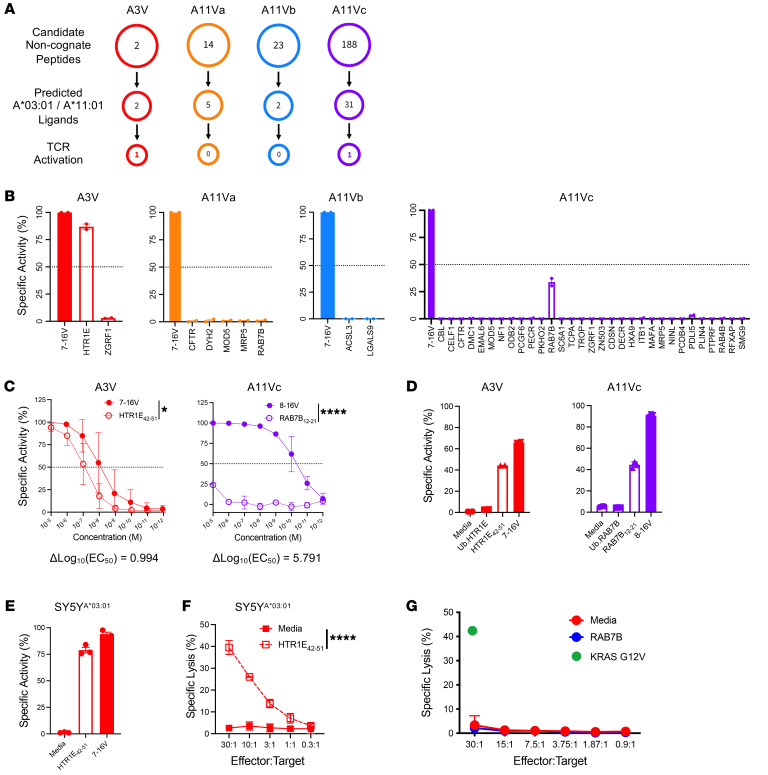
Assessment of TCR crossreactivity to the human proteome. (**A**) Identification of candidate noncognate peptides derived from the human proteome using ScanProsite. HLA-A*03:01 and HLA-A*11:01 ligands identified using NetMHC4.0. Peptides with predicted EC_50_ < 500 nM (NetMHC4.0) were synthesized and screened by in vitro functional assays. (**B**) J^ASP90_C^D8^+^ reporter assay results screening noncognate peptides against A3V (*n* = 2) (red), A11Va (*n* = 5) (orange), A11Vb (*n* = 2) (blue), and A11Vc (*n* = 35) (purple). Cognate KRAS^G12V^ peptide used as positive control (filled bars). (**C**) J^ASP90_C^D8^+^ reporter assay to measure A3V (red) and A11Vc (purple) functional avidity against HTR1E_42–51_ vs 7–16V and RAB7B_12–21_ vs 8–16V, respectively, using HLA-I–matched K562 cells pulsed with titrated levels of peptide. EC_50_ values were determined by nonlinear regression analysis, and differences in TCR functional avidity for crossreactive versus cognate KRAS^G12V^ peptide are displayed as Δlog_10_(EC_50_) values. (**D**) J^ASP90_C^D8^+^ reporter assay to assess A3V and A11Vc reactivity to HLA-I–matched K562 cells alone, endogenously expressing ubiquitinated (Ub) crossreactive protein, or pulsed with crossreactive or cognate KRAS^G12V^ peptide (filled bars). (**E**) J^ASP90_C^D8^+^ reporter assay to assess A3V reactivity to SY5Y^A*03:01^ cells in media alone or pulsed with HTR1E_42–51_ or 7–16V peptide. (**F**) ^51^Cr-release assay evaluating the cytotoxic activity of primary CD8^+^ T cells engineered with A3V against SY5Y^A*03:01^ cells alone or pulsed with HTR1E_42–51_ peptide. (**G**) ^51^Cr-release assay evaluating the cytotoxic activity of primary CD8^+^ T cells engineered with A11Vc against Malme-3M cells alone or pulsed with RAB7B_12–21_ peptide. Cytotoxicity against cognate KRAS 8–16V peptide (30:1 E:T ratio) shown as positive control. Statistical differences between groups were calculated using 2-way ANOVA followed by post hoc pairwise Student’s *t* test with multiple-comparison adjustment. *P < 0.05; ****P < 0.0001.

TCRs may be highly specific yet are inherently degenerate (26). While autoreactive T cells typically undergo negative selection during thymic development, TCR-engineered T cells may demonstrate off-target reactivity to structurally related self-peptides, resulting in severe AEs due to recognition of unrelated peptides (27). Thus, A3V and AV1Va-c recognition motifs generated by X-scan (Supplemental Table 4) served as input for in silico search of potential noncognate/crossreactive peptides encoded by human proteins (Supplemental Data Set 3). Candidate noncognate peptides were curated based on predicted peptide binding to HLA-A*03:01 or HLA-A*11:01 utilizing NetMHC4.0 and setting an affinity threshold of EC_50_ ≤ 500 nM (Figure 3A). TCR-engineered J^ASP90_C^D8^+^ cells were cultured with HLA-I–matched K562 cells pulsed with selected candidate noncognate peptides (A3V, *n* = 2; A11Va, *n* = 5; A11Vc, *n* = 2; A11Vc, *n* = 31) to test for activation (Figure 3B). A3V exhibited reactivity against a peptide (AVIMAIGTTK) derived from amino acid residues 45–51 of 5-hydroxytryptamine receptor 1E, a serotonin receptor encoded by the HTR1E gene (28). A11Vc exhibited weak activity (<50% relative specific activity) to a peptide (IIVGAIGVGK) derived from amino acid residues 12–21 of the Ras-related protein Rab-7b encoded by the RAB7B gene. Both proteins are expressed across a wide array of tissue types, with HTR1E having enriched expression within nervous and ovarian tissues and RAB7B having enriched expression in the skin and proximal gastrointestinal (GI) tract (Human Protein Atlas, https://www.proteinatlas.org/). HTR1E_42–51_ and RAB7_12–21_ are not reported within the HLA Ligand Atlas (29) as HLA-A*03:01– and HLA-A*11:01–restricted epitopes, respectively. We compared the functional avidity of each TCR against these potential crossreactive peptides using TCR-engineered J^ASP90_C^D8^+^ cells. A3V exhibited approximately 10-fold greater functional avidity against 7–16V (EC_50_ = 8.52 nM) versus HTR1E_42–51_ (EC_50_ = 84.02 nM), whereas A11Vc reactivity against RAB7B_12–21_ was weak and limited to high (>10^–5^M) peptide concentrations (Figure 3C).

HLA binding of exogenous peptide does not equate to natural processing and presentation of endogenously expressed antigen. To assess endogenous HTR1E_42–51_ and RAB7B_12–21_ processing and presentation, K562^A03:01^ and K562^A11:01^ cell lines were engineered, respectively, to express the full-length HTR1E or RAB7B proteins tagged with an N-terminal ubiquitination signal (Ub.HTR1E and Ub.RAB7B) to promote proteasomal degradation (Supplemental Figure 3A). In this enhanced proteasomal degradation model, neither A3V- nor A11Vc-engineered J^ASP90_C^D8^+^ cells exhibited reactivity against Ub.HTR1E- or Ub.RAB7B-expressing HLA-I–matched K562 cells (Figure 3D), respectively. We identified cancer cell lines as expressing high levels of HTR1E and RAB7B by transcriptomics, respectively, using the 22Q4 database of the Cancer Dependency Map (DepMap, 2022). SY5Y, a neuroblastoma cell line commonly used to study HTR1E signaling, was engineered to express HLA-A*03:01 (SY5Y^A*03:01^, Supplemental Figure 3B) and validated for HTR1E protein expression by Western blot (Supplemental Figure 3C). A3V-engineered J^ASP90_C^D8^+^ cells cocultured with SY5Y^A*03:01^ did not exhibit TCR reactivity in the absence of exogenous HTR1E_42–51_ or 7–16V peptide (Figure 3E), and primary CD8^+^ T cells engineered to express A3V did not exhibit cytotoxic activity against SY5Y^A*03:01^ cells in 4-hour ^51^Cr-release assays (Figure 3F).Thus, these data suggest that in HTR1E^+^ cells, HTR1E_42–51_ is either not processed or presented at sufficient levels to induce AV3 activation. Malme-3M, a RAB7B^+^ (Supplemental Figure 3E) melanoma cell line, was engineered to express HLA-A*11:01^+^ (Malme-3M^A*11:01^, Supplemental Figure 3D) and tested as a target for endogenous RAB7B_12–21_ presentation. Primary CD8^+^ T cells engineered to express A11Vc did not exhibit cytotoxic activity against Malme-3M^A*11:01^ cells in 4-hour ^51^Cr-release assays (Figure 3G). The lack of A11Vc reactivity with RAB7B_12–21_ peptide at physiologically relevant concentrations (<10^–6^M) or Malme-3M^A*11:01^endogenously expressing RAB7B suggest this TCR reactivity does not present functional crossreactivity at the preclinical development stage. Further safety studies would be required if A3V or A11Vc are to be developed for clinical use.

### TCRs specific for KRAS^G12V^ are of high avidity and exhibit partial CD8^+^ coreceptor dependence.

**Figure 4:**
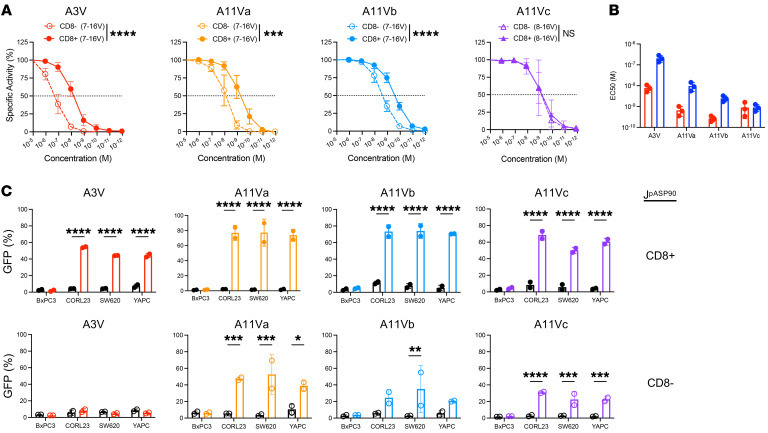
KRAS^G12V^-specific TCRs are of high avidity and exhibit varying degrees of CD8 coreceptor independence. (**A**) Cell-reporter assay using TCR-engineered J^ASP90_C^D8^+^ vs J^ASP90_C^D8^–^ cocultured with HLA-I–matched K562 cells pulsed with titrated levels of cognate 7–16V or 8–16V peptide. Statistical differences between groups calculated using 2-way ANOVA comparing specific activity of J^ASP90_C^D8^+^ versus J^ASP90_C^D8^–^ at 10^–8^ M peptide (A3V) or 10^–9^ M peptide (A11Va-c) followed by post hoc pairwise Student’s *t* test with multiple-comparison adjustment. ***P < 0.001; ****P < 0.0001. (**B**) EC_50_ values of TCR-engineered J^ASP90_C^D8^+^ (red) and J^ASP90–C^D8^–^ (blue) cells as determined by nonlinear regression analysis of data presented in **A**. (**C**) Quantification of NFAT activation (specific activity, %) of TCR-engineered J^ASP90_C^D8^+^ (upper) and J^ASP90_C^D8^+^ (lower) cells following coculture with HLA-I–matched (colored) versus unmatched (black) KRAS^WT^ (BxPC3) or KRAS^G12V^ (CORL23, SW620, YAPC) tumor cell lines. Representative experiments of 2–4 independent evaluations are shown. Statistical differences between groups were calculated using 2-way ANOVA comparing percentages of GFP^+^ TCR-engineered J^ASP90_C^D8^+^ or J^ASP90_C^D8^–^ cocultured with HLA-I matched versus unmatched tumor cells followed by post hoc pairwise Student’s *t* test with multiple-comparison adjustment. *P < 0.05; **P < 0.01; ***P < 0.001; ****P < 0.0001.

TCRs must be of high avidity to detect low levels of pHLA complexes expressed on the surface of tumor cells, and high-avidity TCRs may exhibit CD8-independent activity (30, 31). To assess TCR avidity and CD8 coreceptor independence, we generated KRAS^MUT^ TCR–engineered J^ASP90^ reporter cells that lacked CD8 expression (J^ASP90_C^D8^–^, Supplemental Figure 4A). The functional avidity of each TCR, in the presence or absence of CD8, was assessed by culturing TCR-engineered J^ASP90_C^D8^+^ or J^ASP90_C^D8^–^ cells with HLA-I–matched K562 cells pulsed with titrated 7–16V or 8–16V peptide concentrations (Figure 4A). In the presence of CD8 coreceptor (J^ASP90_C^D8^+^), all TCRs exhibited high antigen avidities to cognate KRAS^G12V^ peptides with EC_50_ values ranging from 7.4 nM (A3V) to 260 pM (A11Vb) (Figure 4B and Supplemental Table 5). In the absence of CD8 coreceptor (J^ASP90_C^D8^–^ cells), A3V, A11Va, and A11Vb exhibited a 1 to 2 log_10_ decreased functional avidity, with A3V being most affected (EC_50_: CD8^+^ = 7.4 nM versus CD8^–^ = 197 nM). In contrast, A11c exhibited no significant functional avidity differences for 8–16V recognition in the presence or absence of the CD8 coreceptor (Figure 4, A and B).

To determine CD8 coreceptor dependency of these TCRs for recognition of endogenously processed and presented 7–16V or 8–16V neoantigens, a panel of KRAS cell lines of various histologic origins engineered to express HLA-A*03:01 or HLA-A*11:01 were tested as targets. TCR-engineered J^ASP90_C^D8^+^ or J^ASP90_C^D8^–^ cells were cocultured with HLA-I–matched or unmatched KRAS^WT^ BxPC3 and KRAS^G12V^ CORL23, SW620, and YAPC cell lines (Figure 4C and Supplemental Figure 4B). A11Va-c displayed partial CD8 coreceptor independent activity, while A3V exhibited complete CD8 dependence for recognition of endogenous antigen. None of the TCRs exhibited reactivity to HLA-I–matched/KRAS^WT^ BxPC3.

### Quantification of 8–16V and 7–16V neoantigen abundance in KRAS^G12V^ tumor cell lines.

To enumerate 8–16V/HLA and 7–16V/HLA complexes expressed by KRAS^G12V^ cancer cell lines, we performed targeted mass spectrometry and absolute peptide quantitation on HLA-A*03:01– or HLA-A*11:01–engineered CORL23, SW620, and YAPC tumor cells. Peptide identity was confirmed by comparing stacked ion fragment intensity (Supplemental Figure 5, A–D) and retention time intensity plots (Supplemental Figure 5, E–H) of eluted versus stable labeled 8–16V or 7–16V peptides. Eluted and internal standard peptide peak area data were used to calculate the abundance of 8–16V and 7–16V epitopes expressed by tumor cells in the context of HLA-A*03:01 or HLA-A*11:01 (Table 2) (32). Enumeration of peptides complexed to HLA-A*03:01 or HLA-A*11:01 identified cell lines with high (CORL23: range 10.4–242.2 complexes/cell), intermediate (YAPC: range 6.0–71.2 complexes/cell), and low (SW620: range 1.6–34.6 complexes / cell) pHLA abundance. We detected higher abundance of 7–16V associated with HLA-A*03:01 and HLA-A*11:01 (range 10.6–242.2 copies/cell) as compared with 8–16V (range 1.6–67.8). Additionally, we detected higher numbers of pHLA complexes for both 7–16V and 8–16V epitopes associated with HLA-A*11:01 (range 4.8–242.2 copies/cell) as compared with HLA-A*03:01 (range 1.6–80.5 copies/cell). Altogether, these data highlight the relative neoantigen/HLA complex abundance by cancer cell lines (<100 pHLA per cell) (15, 33) and the exquisite sensitivity of KRAS^MUT^ TCRs here described.

### TCRs with partial CD8-dependent activity redirect CD4^+^ T cell antitumor effector functions.

**Figure 5:**
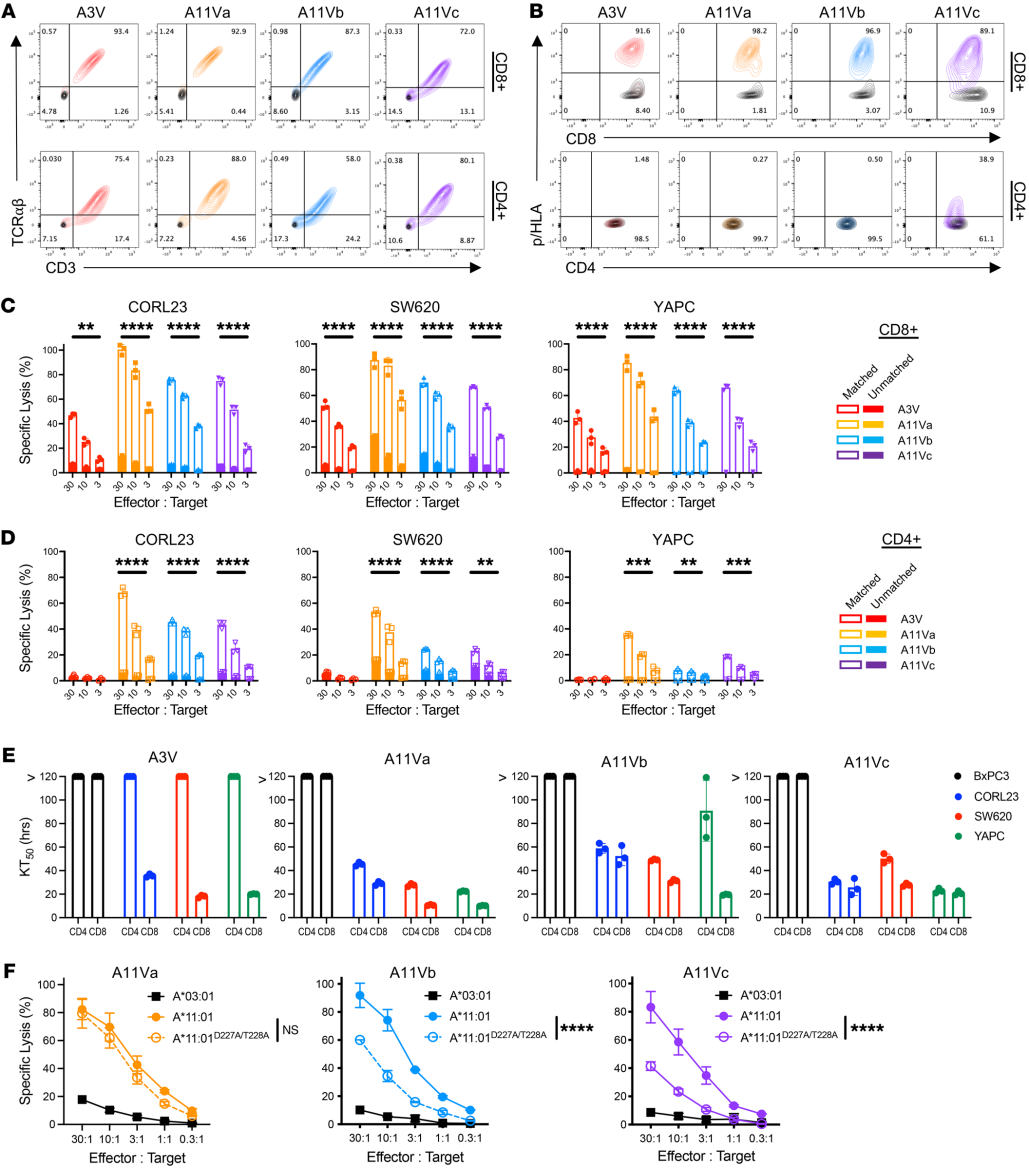
CD4^+^ T cells redirected with partial CD8 coreceptor–independent TCRs exhibit cytotoxic activity. (**A**) FACS plots demonstrating CD3 and TCR-αβ expression by TCR-engineered CD8^+^ (upper) and CD4^+^ (lower) T cells (colored) compared with nontransduced TCR^null^ cells (black). (**B**) FACS plots demonstrating pHLA multimer binding by TCR-engineered (colored) versus TCR^null^ (black) CD8^+^ (upper) and CD4^+^ (lower) T cells. Staining for A11Va-b is shown using 7–16V/ A11:01 multimer, while staining for A11Vc is shown using 8–16V/A11:01. Cytotoxic activity of TCR-engineered (**C**) CD8^+^ and (**D**) CD4^+^ T cells against HLA-I–matched (open) versus unmatched (filled) CORL23, SW620, and YAPC cell lines by 4-hour ^51^Cr-release assay. (**E**) KT_50_, defined as time (hours) to achieved 50% cytolysis at a given E:T ratio, of TCR-engineered CD8^+^ or CD4^+^ T cells against HLA-I–matched BxPC3, CORL23, SW620, and YAPC cell lines by real-time cell analysis. (**F**) Cytotoxic activity of primary CD8^+^ T cells engineered with A11Va, A11Vb, or A11Vc against CORL23 cells expressing HLA-A*11:01^WT^ versus HLA-A*11:01^D227A/T228A^. Statistical comparisons were performed comparing groups at an E:T ratio of 10:1. Statistical differences between groups were calculated using 2-way ANOVA comparing percentages of GFP^+^ TCR-engineered J^ASP90_C^D8^+^ or J^ASP90_C^D8^–^ cocultured with HLA-I–matched versus unmatched tumor cells followed by post hoc pairwise Student’s *t* test with multiple-comparison adjustment. **P < 0.01; ***P < 0.001; ****P < 0.0001.

To assess the antitumor activity of TCR-engineered primary T cells, healthy donor (CD8^+^ and CD4^+^) T cells were lentivirally transduced with KRAS^MUT^ TCRs and CRISPR/Cas9 gene edited to ablate endogenous TCR-α/β expression. Engineered TCR expression was assessed by cell-surface CD3/TCR-aβ expression (Figure 5A) and pHLA multimer staining (Figure 5B). A11Va and A11Vb-engineered CD8^+^ T cells exclusively bound decamer 7–16V/HLA-A*11:01 multimer, while A11Vc-engineered CD8^+^ T exclusively bound nonamer 8–16V/HLA-A*11:01 multimer (Supplemental Figure 6). Upon KRAS^MUT^ TCR expression in CD4^+^ T cells, only A11Vc exhibited weak 8–16V/HLA-A*11:01 multimer reactivity although expression of all TCRs could be validated by anti-pan TCR-αβ staining (Figure 5, A and B). The cytotoxic activity of TCR-engineered CD8^+^ and CD4^+^ T cells was assessed in 4-hour ^51^Cr-release assays against a panel of HLA-I–matched KRAS^G12V^ cell lines. All TCR-engineered CD8^+^ T cells efficiently lysed CORL23, SW620, and YAPC tumor cells (Figure 5C). Additionally, A11Va-c–expressing CD8^+^ T cells exhibited cytotoxic activity against Colo-668, an endogenously (nonengineered) HLA-A*11:01–expressing KRAS^G12V^ cell line (Supplemental Figure 7) (34, 35). Recognition of Colo-668 by A11Va-c CD8^+^ T cells was enhanced by either pretreatment with IFN-γ or lentiviral HLA-A*11:01 overexpression. CD4^+^ T cells expressing A11Va-c exhibited cytotoxic activity against KRAS^G12V^ cell lines, particularly at high effector/target (E:T) ratios (Figure 5D). A3V-engineered CD4^+^ T cells lacked antitumor activity (Figure 5D), a finding consistent with the CD8 coreceptor dependency of this TCR.

Live-cell imaging (Supplemental Figure 8A) and cellular impedance (Supplemental Figure 8B) were employed as complimentary measurements of cell death for visualization of KRAS^MUT^ TCR–mediated lysis over a 72-hour period. Rapid tumor lysis of HLA-I–matched KRAS^G12V^ cell lines without reactivity to KRAS^WT^ was observed for AV11a-c–engineered CD8^+^ T cells with 50% killing (KT_50_) achieved at less than 50 hours (Figure 5E). In contrast, CD4^+^ T cells expressing A11Va-c exhibited slower antitumor activity with KT_50_ ranging from approximately 30 to more than 120 hours (Figure 5E).

To more accurately examine the CD8 coreceptor-dependent activity of A11Va-c, we generated an HLA-A*11:01 single-chain dimer construct in which alanine substitutions were introduced at HLA-A*11:01 residues D227 and T228 (HLA-A*11:01^D227A/T228A^) to abrogate CD8 coreceptor binding (36). CORL23 tumor cells were modified to express HLA-A*11:01^D227A/T228A^ and used as targets in a 4-hour ^51^Cr-release assay. The cytotoxic activity of CD8^+^ T cells expressing A11Va was not impaired by HLA-A*11:01^D227A/T228A^ expression as compared with HLA-A*11:01^WT^, whereas the activity of CD8^+^ T cells expressing A11Vb-c was less efficient (Figure 5F). These findings further support the various degrees of CD8 coreceptor dependency among the 3 G12V/A11:01-specific TCRs.

In addition to direct cytotoxic activity, CD4^+^ T cells engineered with CD8-independent TCRs may secrete soluble factors and cytokines that provide antigen-specific help to enhance the activity and persistence of tumor-specific CD8^+^ T cells (19). We evaluated cytokine expression profiles of TCR-engineered CD8^+^ and CD4^+^ T cells in response to antigen by cytokine bead array and intracellular cytokine staining. TCR-engineered CD8^+^ T cells secreted IL-2, IL-4, IL-6, and IL-10 when cocultured with KRAS^G12V^ tumor cell lines. They also produced IFN-γ and TNF-α, as well as markers of cytotoxic activity including granzyme B and soluble FasL (Supplemental Figure 9A). CD4^+^ T cells expressing A11Va-c exhibited similar expression profiles, whereas A3V-engineered CD4^+^ T cells failed to produce cytokines (Supplemental Figure 9B). By intracellular cytokine staining, both CD8^+^ and CD4^+^ T cells expressing A11Va-c exhibited polyfunctionality with subsets of cells coproducing IFN-γ, TNF-α, and IL-2 upon coculture with CORL23^A*11:01^ cells (Supplemental Figure 9C).

### PAAD patient-derived cell lines are susceptible to KRAS^G12V^-directed TCR-T.

**Figure 6:**
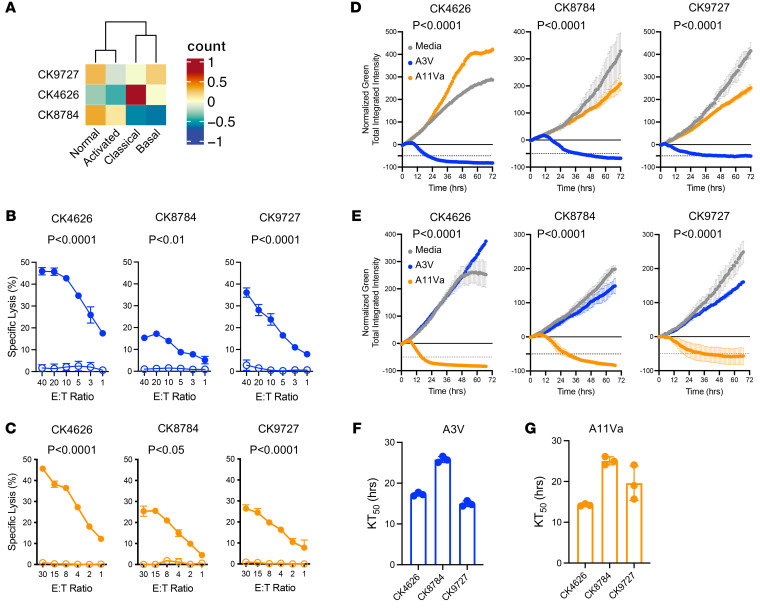
TCR-redirected CD8^+^ T cells exhibit antitumor activity against KRAS^G12V^ PAAD PDCs regardless of molecular subtype. (**A**) Transcriptomic profiling of PDCs. (**B**) Cytotoxic activity by 4h ^51^Cr-release assay of CD8^+^ T cells engineered with TCRA3V against HLA-matched (closed circles) versus mismatched (open circles) PDCs. (**C**) Cytotoxic activity by 4-hour ^51^Cr-release assay of CD8^+^ T cells engineered with TCRA11Va against HLA-matched (closed circles) versus mismatched (open circles) PDCs. (**D**) Cytotoxic activity by real-time cell analysis against A*03:01 expressing PDCs by CD8^+^ T cells expressing TCRA3V versus TCRA11Va. (**E**) Cytotoxic activity by real-time cell analysis against A*11:01 expressing PDCs by CD8^+^ T cells expressing TCRA3V versus TCRA11Va. KT5_0_ values of CD8^+^ T cells expressing (**F**) TCRA3V or (**G**) TCRA11Va against HLA-matched PDCs. Statistical significance indicated; 2-way ANOVA followed by post hoc pairwise Student’s *t* test with multiple-comparison adjustment.

Finally, a panel of KRAS^G12V^ patient–derived cell lines (PDCs, designated as CK4626, CK8784, and CK9727) established from PAAD patient tumor specimens were obtained from the NCI Patient-Derived Models Repository (PDMR) (https://pdmr.cancer.gov/). PDC expression of KRAS^G12V^ was confirmed by next-generation sequencing (NGS) (Supplemental Table 6) and Western blot analysis (Supplemental Figure 10A), and oncogene profiling with *TP53*, *SMAD4*, and *CDKN2A* mutations was consistent with PAAD characteristics. In PAAD, therapeutic sensitivity may be impacted by molecular subtype so the Moffitt molecular classification system was employed to analyze transcriptomic data derived from each PDC, as this classification system is not influenced by stromal components (37–39). RNA-Seq analysis demonstrated each cell line expressed varying transcriptomic profiles with relative enrichment of classical versus basal phenotypes (Figure 6A) (40). Transcriptional phenotyping may be impacted by in vitro expansion of cell lines resulting in transcriptional profiles distinct from that of the original tumor specimen. However, comparison of CK8784 originator versus PDC demonstrated transcriptional clustering, suggesting a degree of PDC transcriptional profile retention in this case (Supplemental Figure 10B).

Both in 4-hour ^51^Cr-release assays (Figure 6, B and C) and long-term (live imaging, Figure 6, D and E) cytotoxicity assays, HLA-I–matched PDCs (Supplemental Figure 10, C and D) are recognized and killed by A3V- and A11Va-engineered CD8^+^ T cells. Determination of KT_50_ values in live imaging assays by nonlinear regression demonstrated 50% tumor elimination between 12 and 24 hours at an E:T cell ratio of 5:1 (Figure 6, F and G). Overall, these data demonstrate PAAD PDCs are susceptible to A3V- and A11Va-mediated killing regardless of molecular subtype.

## Discussion

Neoantigens derived from KRAS^MUT^ restricted to HLA-I and HLA-II alleles have been identified as immunological targets by various genomic and biochemical approaches (41). In this report, data from a phase 1 clinical vaccine trial confirm the immunogenicity of multiple KRAS variants and support the view that KRAS^MUT^ is immunogenic, as mDC vaccination with long peptides elicited T cell immunity, presumably CD4^+^ T cells, in all 5 vaccinated subjects. In contrast, only 2 of 9 subjects administered HLA-I–restricted KRAS^MUT^ short peptide (without immune checkpoint blockade) had detectable CD8^+^ T cell immune responses to the mutant epitope. From subject 2, a TCR A11Vc recognizing the nonamer G12V epitope (VVGAVGVGK) restricted to HLA-A*11:01 was isolated and characterized. From subject 12, immune reactivity to a G12R epitope (EYKLVVVGAR) restricted to HLA-A*33:01/-A33*:03 was detected and is currently under further investigation in our lab. Multiple G12D, G12C, G12V, and G12R HLA-I epitopes were found not to be immunogenic in PAAD patients previously treated with systemic chemotherapy. Interestingly, subject 2 (HLA-A*03:01/A*11:01) preferentially responded to the nonamer G12V epitope when presented by A*11:01 but not A*03:01, which could be explained by the higher affinity of this peptide for A*11:01 versus A*03:01. The focus of this study was the characterization of KRAS^G12V^-specific TCRs restricted to either HLA-A*03:01 or -A*11:01. While all 4 TCRs are of high avidity and recognize human tumor cells in an HLA-restricted manner, the 3 HLA-A*11:01 restricted TCRs (A11Va, A11Vb, and A11Vc) show some degree of CD8 coreceptor independence, in contrast to the HLA-A*03:01–restricted TCR (A3V), which is strictly dependent on the CD8 coreceptor. This feature of CD8 coreceptor independence has important translational significance, since CD4^+^ TCR-T cells demonstrate direct recognition and killing of HLA-I^positive^/HLA-II^negative^ tumor cells, albeit at a slower rate compared with CD8^+^ TCR-T cells (see Figure 5E). Published studies with experimental mouse tumor models (42, 43) as well as CAR-T cell therapies in patients (44) provide compelling evidence supporting a critical role for CD4^+^ T cells in tumor regression.

Small molecule inhibitors of KRAS^MUT^ exhibit highly promising preclinical and clinical results (45), but the durability of clinical responses has been limited by treatment-emergent resistance mechanisms (46–48). Thus, there exists a critical need for the development of alternative, nonredundant strategies to target KRAS^MUT^, and the immune system may play an important role. Recent neoantigen vaccination strategies have demonstrated promising clinical outcomes in patients with resected pancreatic cancer, including a KRAS^G12D/G12R^-targeted amphiphile-based vaccine that primed KRAS^MUT^-specific T cell immunity that correlated with tumor biomarker response and delayed tumor recurrence. However, there remains a limited understanding of minimal KRAS^MUT^ epitopes and their HLA-I–restricting elements that dictate the underlying immune mechanisms important for the generation of CD8^+^ T cell immunity by vaccination (49). A marked limitation of our study is that only 2 vaccine doses were administered to PAAD patients previously treated with systemic combination chemotherapy and the dose/schedule might be suboptimal despite the fact that mature IL-12–producing DCs were employed as adjuvant therapy.

The A3 superfamily of HLA class I alleles is characterized by the F pocket (D77, T80, L81, and D116) showing strong preference for negatively charged residues in the terminal anchor position of the peptide ligand (50). The extended A3 superfamily includes A*11:01, A*29:01, A*30:01, A*31:01, A*32:01, A*33:01, A*74:01, and additional rare alleles that are estimated to provide coverage for 44% of the total US population (13, 14). The KRAS^G12V^ nonamer and decamer epitopes fulfill the allele motif requirement with a terminal lysine and are confirmed experimentally to be high-affinity binders to both HLA-A*03:01 and -A*11:01. HLA-A*33:01 and HLA-A*33:03 share high-sequence homology differing only at amino acid positions 195 (exon 3: α2 domain) and 210 (exon 4: α3 domain). Arginine serves as the preferred terminal anchor residue for the HLA-A33 family of alleles, and KRAS^G12R^ peptide 3-12R (EYKLVVVGAR) with mutant arginine at the terminal position is predicted to bind with high affinity to HLA-A*33:01 as compared with KRAS^WT^ (10.5 nM vs 12132 nM, NetMHC4.0). Studies are ongoing to further evaluate this target using postvaccination blood samples in order to identify TCR clonotypes and validate epitope presentation. Based on our calculations, it is estimated that 5,770 newly diagnosed cancer patients per year in the US would be eligible for G12V/A*11:01-directed TCR-T, while 8,655 patients would be eligible for G12V/A*03:01-directed TCR-T therapy (41). In our study, vaccinated HLA-A*02:01^+^ patients were unable to generate CD8^+^ T cell immunity to the native or spliced G12V variant peptide (Supplemental Table 3). This void necessitates an appreciation that KRAS^MUT^-directed immunotherapies will require personalization (HLA matching with KRAS variant) and that not all patients would be ideal candidates.

TCRs generally exhibit reactivity to HLA-I–restricted peptides of one length (51), yet we found that A11Vc derived from a vaccinated patient exhibited reactivity to both nonamer (8–16V) and decamer (7–16V) peptides. However, pHLA multimer assays showed no binding of A11Vc to the 7–16V/HLA-A*11:01 multimer, yet strong binding to the 8–16V/HLA-A*11:01 multimer, consistent with stringent recognition of the nonamer epitope. Interestingly, TCR recognition motif (“TCR footprint”) assays showed that P1 of the decamer sequence (V/VVGAVGVGK) was dispensable for A11Vc TCR recognition, suggesting that the amino terminal residue was cleaved by an aminopeptidase to generate a high-affinity nonamer bound to HLA-A*11:01. Immuno-peptidomic analysis has confirmed presentation of both the decamer and the nonamer length epitopes (15); therefore, we hypothesize that A11Vc has strict specificity for the nonamer epitope bound to HLA-A*11:01.

Quantitative immunopeptidomics to assess antigen density on KRAS^G12V^ human tumor cell lines demonstrates, in most cases, fewer than 100 pHLA complexes per cell, which highlights the sensitivity of natural TCRs restricted to the A3 superfamily, most notably HLA-A*11:01. The data reveal low-level expression of both nonamer and decamer epitopes ranging from a high of 242 decamer (VVVGAVGVGK) complexes on COR-L23^A*11:01^ to a low of 1.6 nonamer (VVGAVGVGK) complexes on SW620^A*03:01^. One consistent observation noted by several groups is that the decamer epitope appears to be the predominant peptide species present in tumor cells (15, 33). Of note, immunopeptidomic analysis of COLO668 cells positively identified the decamer 7–16V peptide (34, 35), but not the nonamer species, suggesting the abundance of endogenous 8–16V/A*11:01 pHLA complexes in this cell line may be below the limit of detection of A11Vc TCR-T cells (see Supplemental Figure 7C). This example highlights a primary limitation of TCR-T cell therapies, which require tumor cell-surface expression of target antigen (pHLA) density above a certain threshold to allow for effector cell recognition.

Our report provides an in-depth functional assessment of KRAS^MUT^ TCRs emphasizing recognition of the G12V neoantigen. We compare TCRs identified from both vaccinated subjects and healthy volunteers to identify those with the following properties: (a) high specificity for a bona fide tumor neoantigen, (b) sufficient avidity to detect endogenous antigen expression by tumor cells, and (c) no discernible crossreactivity to other peptides encoded within the human proteome. Among a panel of 4 TCRs specific for neoantigens of KRAS^G12V^ restricted to HLA-A*03:01 or HLA-A*11:01, we identify TCR A11Va as a lead candidate for clinical development for the treatment of HLA-A*11:01^+^ patients with advanced KRAS^G12V^ solid tumors. Based upon the allele frequency of HLA-A*11:01 in the US population and the prevalence of KRAS^G12V^ among PAAD, COAD, and LUAD patients, we estimate that A11Va TCR-T therapies could be applicable to more than 5,000 patients per year in the USA (41).

## Methods

### Sex as a biological variable.

Our study examined male and female patients, both men and women were eligible for trial, and findings were similar for both.

### Clinical trial.

A pilot clinical study was designed to assess the safety, tolerability (primary endpoint), and immunological outcomes (secondary endpoint) of an autologous mDC vaccine targeting mutant KRAS (mDC3/8-KRAS) and conducted at the Abramson Cancer Center and the Hospital of the University of Pennsylvania (ClinicalTrials.gov NCT03592888). Eligible patients included adults with a history of locoregional pancreatic adenocarcinoma treated with no more than 2 lines of neoadjuvant or adjuvant chemotherapy and surgery without evidence of disease recurrence (ECOG performance status 0–1). Additional eligibility criteria included a pathologically confirmed KRAS^G12^ mutation and expression of one or more of the following HLA-I alleles: HLA-A*02:01, HLA-A*03:01, HLA-A*11:01, HLA-B*07:02, and HLA-C*08:02.

A leukapheresis was performed to obtain PBMCs from patients through the University of Pennsylvania Clinical Cell and Vaccine Production Facility prior to dose 1 (prime) and after dose 2 (boost), and PBMCs were collected weekly (prior to vaccination and until week 12) to assess the kinetics of immune responses. All mDC3/8-KRAS vaccine doses were prepared at the time of immunization from either freshly isolated (prime) or cryopreserved (boost) PBMCs (all derived from the same leukapheresis collection) as previously described (22). Two hours prior to infusion, mDC3/8 were pulsed (50 μg/10^6^ cells/ml) separately with 1–7 peptides, separately or pooled in select instances. For priming dose, influenza virus vaccine (Sanofi Pasteur) was added to provide a source of recall antigen for CD4^+^ T cells. mDC3/8-KRAS infusions were given i.v. in the outpatient clinic for a total of 2 doses 8 weeks apart. For priming dose, patients received 3.0 × 10^7^ DCs per peptide (up to 2.1 × 10^8^ DCs total); for booster dose, patients received 1.5 × 10^7^ DCs per peptide (up to 1.05 × 10^8^ DCs total). Peptides were obtained lyophilized (>95% purity; Bachem) and dissolved in 1% DMSO in sterile water, then tested for sterility, purity (residual solvent), and endotoxin. For select peptides, binding assays were performed (Pure Protein, LLC) to confirm HLA-I binding. For infusion, mDC3/8-KRAS was resuspended in 50 ml normal saline supplemented with 5% human serum albumin and administered over 30 minutes by i.v. infusion after premedication with 650 mg acetaminophen.

Patients underwent clinical evaluation prior to each mDC3/8-KRAS infusion. Toxicities and adverse effects were graded according to the National Cancer Institute Common Terminology Criteria for Adverse Events (CTCAE, version 5.0; https://ctep.cancer.gov/protocoldevelopment/electronic_applications/docs/CTCAE_v5_Quick_Reference_5x7.pdf).

### Immune assessment.

Immunologic analysis to evaluate the kinetics and magnitude of T cell response to mutant KRAS^G12^ peptides upon vaccination was performed using PBMCs collected at defined time points (prior to vaccination and up to week 12). Fresh PBMCs obtained by Ficoll-Hypaque gradient centrifugation were adjusted to 2 × 10^6^ cells/ml in Optimizer T Cell Expansion Media (Thermo Fisher Scientific) containing 5% human AB-serum and dispersed at 1 ml/well in 24-well plates. Cultures were set up for the mKRAS^G12^ peptides (short/long peptides) and positive peptide control peptides (data not shown). Cultures were pulsed with 40 μg/ml peptide and 50 U/ml IL-2 fed starting at 48 hours and every other day thereafter. On day 14, cultures were harvested, counted, and analyzed by IFN-γ ELISPOT analysis as previously described.

Final immune assessment was performed using purified CD8^+^ T cells isolated from pre- and postvaccine leukapheresis samples. PBMCs were isolated from leukapheresis samples by Ficoll-Hypaque gradient centrifugation, and CD8^+^ T cells were isolated using a CD8 Negative Selection Kit (Miltenyi Biotech). Purified CD8^+^ T cells were cultured at a 20:1 ratio with irradiated (2,500 rads) autologous mDC3/8 pulsed with peptide (40 μg per 1 × 10^6^ DC / mL) in 24-well trays in Optimizer CST media (Gibco; Thermo Fisher Scientific) supplemented with 5% pooled human sera. Cell culture media was supplemented with 50 U/mL IL-2 (Chiron) starting at day 2, then every 48 hours following secondary stimulation. On day 14, antigen-specific T cell responses were identified by pHLA multimer staining and flow cytometry, and TCR sequences were identified as previously described.

### Primary cells.

PBMCs and purified CD4^+^ and CD8^+^ T cells were provided by the University of Pennsylvania Human Immunology Core after cell isolation from apheresis products of HLA class I– and class II–typed healthy donors.

### Cell lines.

The following cell lines were cultured in RPMI media supplemented with 10% FBS, 2 mM l-glutamine (Corning, G 25-005-CI), and 1× penicillin/streptomycin (Corning, G 30-002-CI): K562 (ATCC, CCL-243), Jurkat E6-1 (ATCC, TIB-152), BxPC-3 (ATCC, CRL-1687), COR-L23 (Sigma-Aldrich, 92031919), SW620 (ATCC, CCL-227), YAPC (DSMZ, ACC 382), Malme-3M (ATCC, HTB-64) and Colo668 (MilliporeSigma 87061209). SY5Y cells (provided by Michael Milone, University of Pennsylvania, Philadelphia, Pennsylvania, USA) were cultured in RPMI media supplemented with 20% FBS and l-glutamine.

### PDCs.

The following PDCs used in this study were developed by the NCI PDMR, Frederick National Laboratory for Cancer Research: CK4626 (K24384-001-R-PDC), CK8784 (485368-065-R4-J2-PDC), and CK9727 (561559-040-R-J1-PDC). PDCs were grown in DMEM/F12 media (Invitrogen 12634-010) supplemented with 10% FBS (Hyclone, SH30070.03H1), hydrocortisone (Sigma H4001), EGF recombinant human protein (Invitrogen PHG0311), Aadenine (MilliporeSigma A2786), penicillin/streptomycin (Invitrogen, 1514022), l-glutamine (Invitrogen, 25030-081), and Y-27632 dihydrochloride (Tocris Bioscience 1254), as specified by PDMR SOP30101.

Information regarding PDC mutational profiles was extracted from PDMR whole-exome sequencing data as reported in the following PDMR databases: K24384~001-R~PDC~v2.0.1.50.0, 485368~065-R4~J2-PDC~v2.0.1.51.0, and 561559~040-R~J1-PDC~v2.0.2.51.0.

PDC transcriptional profiling was performed using the following RNA-Seq files obtained from the PDMR database: K24384~001-R~PDC~v2.0.1.4.0, 485368~065-R4~J2-PDC~v2.0.1.4.0, and 561559~040-R~J1-PDC~v2.0.2.4.0. Transcriptional profiling of CK8784 originator tumor specimen was performed using the 485368~065-R4~ORIGINATOR~v2.0.2.11.0 RNA-Seq file. Gene set analyses were performed using the GSVA R package with default parameters.

### Peptides and biochemical peptide-HLA binding assays.

Custom peptides were synthesized by BACHEM or Proimmune to greater than 95% purity. Lyophilized peptides were dissolved in 10% DMSO (Mylan Cryoserv, 67457-178-50) in sterile water pH 7.4 and passed through a 0.2 μM Centrex filter (10467013). Positional scanning peptide library peptides were synthesized by JPT Peptide Technologies to greater than 70% purity and resuspended in 100% DMSO. Analysis of pHLA-binding affinity and stability data was performed on previously published results.

### Antibodies and flow cytometry.

The following primary antibodies along with vendor, catalog number, and working dilution used for flow cytometry in this manuscript are as follows: anti-human HLA-A, B, C–APC (W6/32) (BioLegend, 311410, 1:50), anti-human HLA-A3–PE (GAP.A3) (eBioscience, 12-5754-42, 1:20), anti-human HLA-A11–Biotin (One Lambda, BIH0084, 1:20), anti-human CD3–PE (Invitrogen, MHCD0304, 1:100), anti-human CD4–BV421 (OKT4) (BioLegend, 317434, 1:100), anti-human CD8–APC (SK1) (BioLegend, 344722, 1:100), and anti-human TCR-αβ–APC (IP26) (BioLegend, 306717, 1:20). The following custom pHLA multimers along with vendor and working dilution information were used: VVVGAVGVGK/HLA-A*03:01–biotin (Proimmune, custom, 1:100), VVVGAVGVGK/HLA-A*11:01–PE (Immudex, custom, 1:100), and VVGAVGVGK/HLA-A*11:01–PE (Immudex, custom, 1:100).

Intracellular cytokine staining was performed using the Cytofix/Cytoperm Plus kit (BD Biosciences) following the manufacturer’s protocol. Briefly, CD4^+^ and CD8^+^ T cells expressing A11Va, A11Vb, or A11Vc were mixed with CORL23-A11 tumor cells at an E:T ratio of 3:1 and incubated for 4 hours at 37°C in the presence of GolgiPlug (BD Biosciences). After staining for cell-surface molecules, cells were fixed, permeabilized, and stained with the following antibodies: anti-human IFN-γ–PE (BioLegend, 502509, 1:50), anti-human TNF-α–BV650 (BioLegend, 502938, 1:50), and anti-human IL2–APC (BioLegend, 500310, 1:50). Data were analyzed using Simplified Presentation of Incredibly Complex Evaluations (SPICE, version 6.1).

Cells were stained at the specified antibody dilutions and washed in FACS buffer. Data were acquired with a BD LSRFortessa flow cytometer using BD FACSDiva software (version 8.0.2) and analyzed using FlowJo software (version 10.8.1). For rapid expansion of sorted T cells, anti-human CD3 (OKT3) (LEAF, 317326, 30 ng/ml) was used.

### Cytokine multiplex assay.

CD8^+^ or CD4^+^ T cells expressing A3V, A11Va, A11Vb, or A11Vc were resuspended at 1 × 10^6^ cells/mL in tumor growth media containing 3.33 1 × 10^5^ tumor cells for an E:T ratio of 3:1 and plated into 48-well tissue culture plates. Conditioned media was harvested 24 hours later, centrifuged to remove debris, and stored at −80°C. Conditioned media were analyzed using the LEGENDplex Human CD8/NK Panel (BioLegend, 740267) according to the manufacturer’s guidelines.

### Lentiviral constructs and production.

All lentiviral constructs were generated as previously reported using the third-generation lentiviral transfer vector pTRPE-EGFP-T2A-mCherry (provided by Michael Milone, University of Pennsylvania, Philadelphia, Pennsylvania, USA). HLA-A*03:01, HLA-A*11:01, HLA-A*11:01^D227A/T228A^, HLA-A*33:01, and HLA-A*33:03 single-chain dimer constructs were PCR amplified from corresponding plasmids and used to replace the mCherry moiety on the pTRPE-EGFP-T2A-mCherry vector backbone. Synthetic TCR DNA vector constructs were synthesized (TWIST Bioscience) as previously described (15) to include TCR-α and TCR-β chains separated by a T2A sequence. TCR-α and TCR-β constant domains were codon altered to be resistant to Cas9 protein riboprobes targeting endogenous *TRAC* and *TRBC1/TRBC2*. Ub.HTR1E was generated from a synthetic construct encoding the full-length HTR1E protein with a 5′ubiquitin tag as previously described (15). The DNA construct was subcloned into the EGFP moiety of the pTRPE-EGFP-T2A-mCherry vector for the generation of lentivirus. Viral particles were used to engineer K562-A3 cells, which were sorted to purity based on mCherry expression.

### Jurkat reporter system to assess TCR antigen specificity and avidity.

Sorted A3V and A11Va-c J^ASP90_C^D8^+^ or J^ASP90_C^D8^–^ cell lines were mixed at a 1:1 ratio with HLA-SCD expressing K562 cells pulsed with titrated peptide concentrations (10 μM–1 pM). After 16 hours, cells were analyzed by flow cytometry to determine the percentage of EGFP^+^ cells in each sample. Cells activated with PMA (50 ng/ml) and ionomycin (750 ng/ml) were included as positive controls. Reporter cells cultured with 10 μM peptide-pulsed K562 cells were used for maximal activation (%GFPmax), and reporter cells cultured in media alone were used as negative controls (%GFP_Min_). Specific activity (%) in Jurkat assays was calculated by the following equation: (%GFP_Test_ – %GFP_Min_)/(%GFP_Max_ – %GFP_Min_) × 100. Data were fitted to a dose-response curve by nonlinear regression analysis ([agonist] vs. normalized response) to determine EC_50_ values using GraphPad Prism, version 9.2.0.

### Proteomic quantitation of KRAS^G12V^ peptides presented by human tumor cells.

Single-cell clones were isolated from previously described HLA-SCD–engineered CORL23, SW620, and YAPC tumor cell lines (15). CORL23-A3, CORL23-A11, SW620-A3, SW620-A11, YAPC-A3, and YAPC-A11 clonal cell lines were expanded to 1–2 × 10^8^ total cells. HLA class I IP was performed by Cayman Chemical as previously described (15). DDA analysis of cell lines was performed by MS Bioworks using 50% of the enriched sample. The quantification of 8–16V and 7–16V epitopes expressed by HLA-engineered KRAS^G12V+^ cell lines was performed by targeted MS using PRM (MS Bioworks). Peptides were enriched as described above. Synthetic stable labeled peptides VVGAVGVGK^ and VVVGAVGVGK^ were purchased from New England Peptide, where ^ is lysine (^13^C_6_^15^N_2_). In all, 200 fmols of each stable labeled peptide was added to the enriched samples for analysis. Each enriched sample was analyzed in duplicate (50% of the sample per injection). PRM was performed with a Waters M-Class HPLC system interfaced to a Thermo Fisher Fusion Lumos mass spectrometer. Peptides were loaded on a trapping column and eluted over a 75 μm analytical column at 350 nl/min; both columns were packed with Luna C18 resin (Phenomenex). The mass spectrometer was operated in PRM mode with the Orbitrap operating at 17,500 FWHM resolution. Collision-induced dissociation data were collected for the (M+2H)2+ charge state ions of the target peptides VVGAVGVGK, VVGAVGVGK^, VVVGAVGVGK, and VVVGAVGVGK^. Extracted ion chromatograms for each of the target peptides were generated manually using XCalibur QualBrowser software, version 4.1.31.9 (Thermo Fisher), with a 20 ppm mass tolerance for product ions. For the calculation of 8–16V and 7–16V epitopes expressed by HLA-A*03:01 and HLA-A*11:01 complexes, eluted and internal standard peptide peak area data were used to calculate the number of moles of peptide present in the sample. This number was doubled (half the IP was analyzed in a single injection) and converted to molecules by multiplying by Avogadro’s number. The result was divided by the number of input cells to give number of peptide molecules/cell. Peptide identity was confirmed by comparing the retention time and stacked ion fragment intensity plots of eluted versus stable labeled 8–16V or 7–16V peptides.

### Combinatorial peptide library scan for determination of TCR recognition motifs.

Sorted A3V and A11Va-c J^Asp90_C^D8^+^ cell lines were mixed 1:1 with HLA-I–matched K562 cells pulsed with 10 μM peptide. After 16 hours, EGFP expression was assessed by FACS to measure the percentage of activated J^Asp90_C^D8^+^ cells. Percentage of specific activity was calculated by standardizing activation via the cognate peptide (VVVGAVGVGK) and no peptide to 100% and 0%, respectively. Calculated specific activity values exceeding 100% were trimmed to 100%, and negative values were adjusted to zero. The recognition patterns of A3V and A11Va-c were illustrated as both heatmaps and Seq2Logo graphs. Seq2Logo plots depict only amino acids at each position with a minimum 50% specific activity value using the PSSM-Logo algorithm (52).

### Computational prediction of noncognate peptides.

A noncognate peptide reactivity search was performed against the UniProtKB Human Protein Database using ScanProsite (53) using binding motifs with a 50% specific activity value as a cutoff; the match mode was greedy, overlaps, no includes. Predicted binding of the identified peptides to the corresponding HLA-I (HLA-A*03:01 or HLA-A*11:01) was determined with NetMHC, version 4.0 (https://services.healthtech.dtu.dk/services/NetMHC-4.0/). Identified peptides fitting the binding motif pattern were synthesized with a predicted affinity of 500 nM or “weak binder” as a cutoff.

### Western blot for validation of KRAS, HTR1E, and RAB7B expression.

Cell lines were lysed in RIPA buffer, incubated on ice for 30 minutes, and centrifuged at 14,000*g* for 10 minutes in a refrigerated centrifuge. Cell lysates were collected in fresh tubes and placed on ice. Protein concentration was quantified using Pre-Diluted Protein Assay Standards: BSA Set (Thermo Scientific, catalog 23208) and DC Protein Assay (Bio-Rad, catalog 5000112) according to the manufacturer’s instructions. 4× NuPAGE Loading Buffer (Invitrogen, catalog NP0007) and 10× NuPAGE Reducing Agent (Invitrogen, catalog NP0009) were added to 40 μg of protein and boiled at 95°C for 10 minutes. For analysis of mutant-specific KRAS^G12V^ and RAB7B, 200 μg of protein was analyzed. Samples were loaded onto 4%–12% NuPAGE Bis-Tris 1.5 mm gels (Invitrogen, catalog NP0335) using the XCell SureLock Mini-Cell (Invitrogen, catalog EI0001) and semi-dry transferred to a methanol-activated PVDF membrane (Bio-Rad, catalog 1620177) in the Invitrogen Power Blotter XL System (Invitrogen, catalog PB0013). Membranes were blocked in Intercept (TBS) blocking buffer (LI-COR, catalog 927-60001). Membranes were incubated overnight at 4°C with the following primary antibodies diluted in Intercept antibody diluent (LI-COR, catalog 927-650001): rabbit anti-human HTR1E (1:1000, DCABH-15693, Creative Diagnostics), mouse anti-human KRAS (clone 2C1) (1:1000, LSBio, catalog LS-C175665), rabbit anti-ras (G12V Mutant Specific) (1:125, Cell Signaling, catalog 14412), mouse anti-RAB7B (Abnove, catalog H00338382-M01), or mouse anti-human B-actin (1:1000, Cell Signaling Technology, catalog 8H10D10). The membranes were washed with Tris-buffered saline with 0.1% Tween (TBST) and incubated with LI-COR IRDye 680RD goat anti-rabbit IgG secondary antibody (1:10,000, LI-COR, catalog 926-68071) or LI-COR IRDye 800CW goat anti-mouse IgG secondary antibody (1:10,000, LI-COR, catalog 926-32210). The membranes were washed again with TBST and kept in TBST until imaging. Membranes were imaged with the Odyssey CLx Infrared Imaging System (LI-COR, catalog 46677). When membranes were reused to evaluate expression of additional proteins, they were first stripped in Restore Western Blot Stripping Buffer (Thermo Scientific, catalog 21059) and then processed as previously described.

### ^51^Cr-release assay.

Tumor cell lines were labeled with 25 μCi ^51^Cr in the presence or absence of peptide (10 μM) for 1 hour at 37°C, washed, and tested as targets in a standard 4-hour 51Cr-release assay. Effector cells consisted of primary gene-edited (TCR-αβ^null^) CD8^+^ or CD4^+^ T cells engineered with synthetic TCRs specific for KRAS^G12V^, designated A3V, A11Va, A11Vb, and A11Vc. Transgenic TCR expression was assessed by FACS analysis and pHLA multimer binding. Assays were performed, in triplicate, at various E:T ratios. Data were collected using a MicroBeta2 LumiJET Microplate Counter (PerkinElmer). Data are represented as percentage of specific lysis reported as mean ± SD. Specific lysis (%) of target cells was calculated by the following equation: (test – min)/(max – min) × 100

### Real-time apoptotic cell-death analysis.

Real-time apoptotic cell-death analysis (live cell imaging with cellular impedance) was performed to assess extended cytotoxic activity using the xCELLigence Real Time Cell Analysis eSight system (ACEA Biosciences). Target tumor cells were plated (1 × 10^4^ cells/well) and allowed to adhere for 24 hours. Effector T cells were added at an E:T ratio of 3:1. Time-lapse video monitoring was performed with acquisition of brightfield and green (GFP) every hour for 72 hours. Concurrent cell index (relative cell impedance) was monitored every 15 minutes. Data were normalized to the maximum total integrated intensity or cell index value immediately following effector-cell plating. Shaded lines reflect the mean of replicate wells ± SD. The amount of time required to kill 50% of target tumor cells (KT_50_) was determined by nonlinear regression analysis using GraphPad Prism, version 9.2.0.

### Statistics.

GraphPad Prism (RRID:SCR_002798), version 9.2.0, was used for statistical analyses and graphical representation. Data are presented as means ± SD or SEM. Statistical analysis of multiple comparisons was performed using a 2-way ANOVA with Šidák’s or Tukey’s HST post test, and comparisons between just 2 groups were performed using Student’s unpaired *t* test. Significance of overall survival was determined via Kaplan-Meier analysis with log-rank (Mantel-Cox) analysis. The significance threshold was set to α_Test_ = 0.05. To account for multiple comparisons, the Bonferroni-corrected α (α_Bonferroni_) was determined by dividing α_Test_ by the total number of comparisons (*n* = 10), establishing a significance threshold of α_Bonferroni_ = 0.005. For EC_50_ generation, data were normalized, and agonist versus response test was used with a nonlinear regression model. All data presented are representative of 2 or more independent experiments.

### Study approval.

This study was approved by the Institutional Review Board at the University of Pennsylvania (IRB# 830261). Healthy donors were enrolled on the Institutional Review Board–approved research protocol 705906 at the University of Pennsylvania. All patients provided written, informed consent for the clinical study. The FDA approved procedures for DC manufacturing and administration according to BB-IND 18328. The study protocol is available as a supplemental file associated with this manuscript.

### Data availability.

Values for all data points in graphs are reported in the Supporting Data Values file. Additional information can be obtained from upon request.

## Author contributions

ASB, RHV, GPL, and BMC conceived the project. ASB, RBN, AJR, GPL, BMC, and MJF selected the methodology. AJR developed the software. KLS, TB, MHE, CX, RJS, and MLB performed the validation experiments. ASB, RBN, MJF, and BMC performed the formal analysis. RJ performed formal analysis and visualization. DJP, BMC, GPL, and RHV provided resources. ASB, AJR, MLB, and BMC curated data. ASB, RBN, RHV, GPL, and BMC wrote the original draft of the manuscript. ASB, MHO, RHV, GPL, and BMC reviewed and edited the manuscript. GPL and BMC supervised the project. RHV, GPL, and BMC acquired funding.

## Supplementary Material

Supplemental data

ICMJE disclosure forms

Supplemental data sets 1-3

Unedited blot and gel images

Supporting data values

## Figures and Tables

**Table 2:**
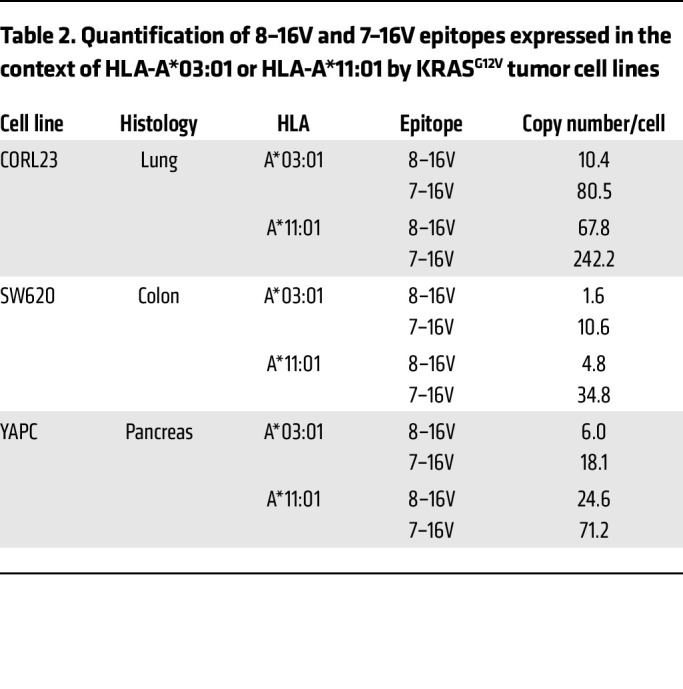
Quantification of 8–16V and 7–16V epitopes expressed in the context of HLA-A*03:01 or HLA-A*11:01 by KRAS^G12V^ tumor cell lines

**Table 1:**
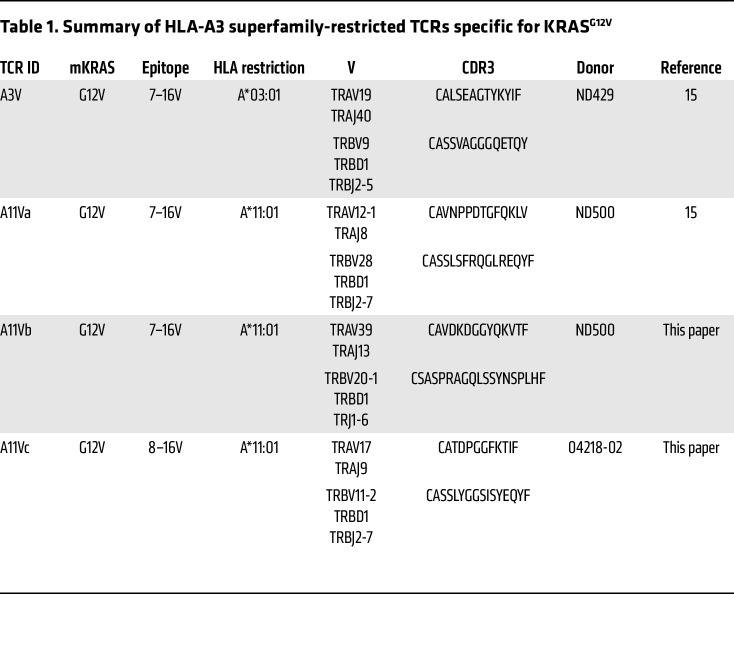
Summary of HLA-A3 superfamily-restricted TCRs specific for KRAS^G12V^

## Version 1 09/17/2024

In-Press Preview

## Version 2 11/01/2024

Electronic publication
