# Supplementary material for: Natural TCRs targeting KRASG12V display fine specificity and sensitivity to human solid tumors
Source: J Clin Invest. 2024 Sep 17;134(21):e175790. doi: 10.1172/JCI175790 (PMC11529987; doi:10.1172/JCI175790)
Supplement: Supplemental data [file jci-134-175790-s009.pdf]

Bear et al. Supplemental Figures and Tables

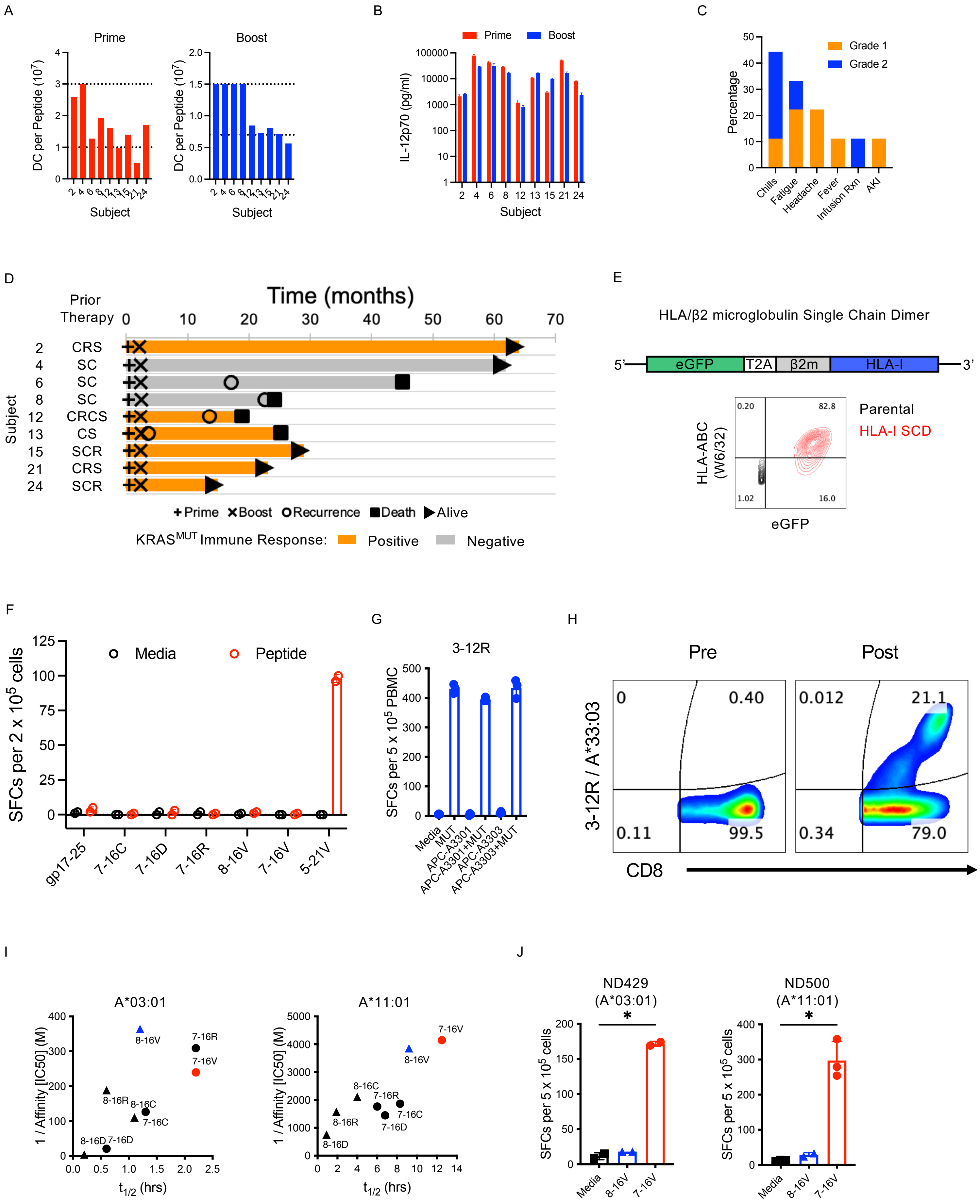
A
Prime
Boost
DC per Peptide (10^7)
Subject
B
Prime
Boost
IL-12p70 (pg/ml)
Subject
C
Grade 1
Grade 2
Percentage
Chills
Fatigue
Headache
Fever
Infusion Rxn
AKI
D
Time (months)
Prior Therapy
Subject
CRS
SC
SC
SC
CRCS
CS
SCR
CRS
SCR
+Prime ×Boost ORecurrence ■Death ▶Alive
KRAS^MUT Immune Response: Positive Negative
E
HLA/β2 microglobulin Single Chain Dimer
5'
eGFP
T2A
β2m
HLA-I
3'
Parental
HLA-I SCD
HLA-ABC (W6/32)
eGFP
0.20
82.8
1.02
16.0
F
Media
Peptide
SFCs per 2 x 10^5 cells
gp17-25
7-16C
7-16D
7-16R
8-16V
7-16V
5-21V
G
3-12R
SFCs per 5 x 10^5 PBMC
Media
MUT
APC-A3301
APC-A3301+MUT
APC-A3303
APC-A3303+MUT
H
Pre
Post
3-12R / A*33:03
CD8
0
0.40
0.11
99.5
0.012
21.1
0.34
79.0
I
A*03:01
A*11:01
1 / Affinity [IC50] (M)
t1/2 (hrs)
8-16V
7-16R
7-16V
8-16R
8-16C
7-16C
8-16D
7-16D
J
ND429 (A*03:01)
ND500 (A*11:01)
SFCs per 5 x 10^5 cells
Media
8-16V
7-16V
*

Supplemental Figure 1: Feasibility, safety, and immunogenicity of mDC3/8-KRAS vaccination.

(A) Bar graphs representing vaccine dose presented as the number of dendritic cells per peptide administered to each subject. Prespecified dose ranges for Prime (10-30 x 10^6 DC/peptide) and Boost (7-15 x 10^6 DC/peptide) are indicated by dashed lines.
(B) ELISA assay results of IL-12p70 concentration in the media supernatant of autologous dendritic cells plated at 1 x 10^6 DCs/ml following 24 hr maturation.
(C) Frequency and grade of AEs attributable to mDC3/8-KRAS vaccination.
(D) Swimmer plot of outcomes of 9 vaccinated subjects from time of study eligibility confirmation. Prior sequential standard of care therapy for each subject is indicated: C, chemotherapy; S, surgery; R, radiation.
(E) Schematic representation of synthetic HLA/β2 microglobulin Single Chain Dimer construct and FACS representation of K562 cells engineered with lentiviral particles encoding HLA-I SCD (Red) vs parental (Black) cells.
(F) Assessment of Subject 04218-24 HLA-I restricted T cell responses against HLA-A*03:01 targeted control (gp17-25) and $KRAS^{MUT}$ peptides compared to HLA-II targeted 5-21V long peptide. IFN-γ ELISpot assay following ex vivo expansion of Week 3 post-vaccine PBMC comparing cells cultured in media alone (Black) compared to in the presence of target peptide (Red).
(G) Assessment of Subject 04218-12 HLA-I restricted T cell responses against 3-12R peptide by IFN-γ ELISpot assay following ex vivo expansion of Week 3 post-vaccine PBMC. Monoallelic K562 cells expressing HLA-A*33:01 (APC-A3301) or HLA-A*33:03 (APC-A3303) were used to identify HLA-I restriction. MUT indicates mutant KRAS peptide.
(H) pHLA-multimer analysis to assess CD8+ T cell response against 3-12R/A*33:03 following in vitro expansion of pre- (Week -1) and post-vaccine (Week 3) PBMC.
(I) Peptide-HLA affinity and stability measurements of 9-mer (8-16) and 10-mer (7-16) mutant KRASG12 epitopes (G12C, G12D, G12R, G12V) complexed with HLA-A*03:01 or HLA-A*11:01.
(J) IFN-γ ELISPOT assay results following in vitro expansion of healthy donor purified CD8+ T cells stimulated with 8-16V or 7-16V peptide-pulsed autologous matured dendritic cells. IFN-γ ELISPOT Spot Forming Cell (SFC) values in response to 8-16V (blue) or 7-16V (red) are compared to CD8+ T cells cultured in media alone (black).

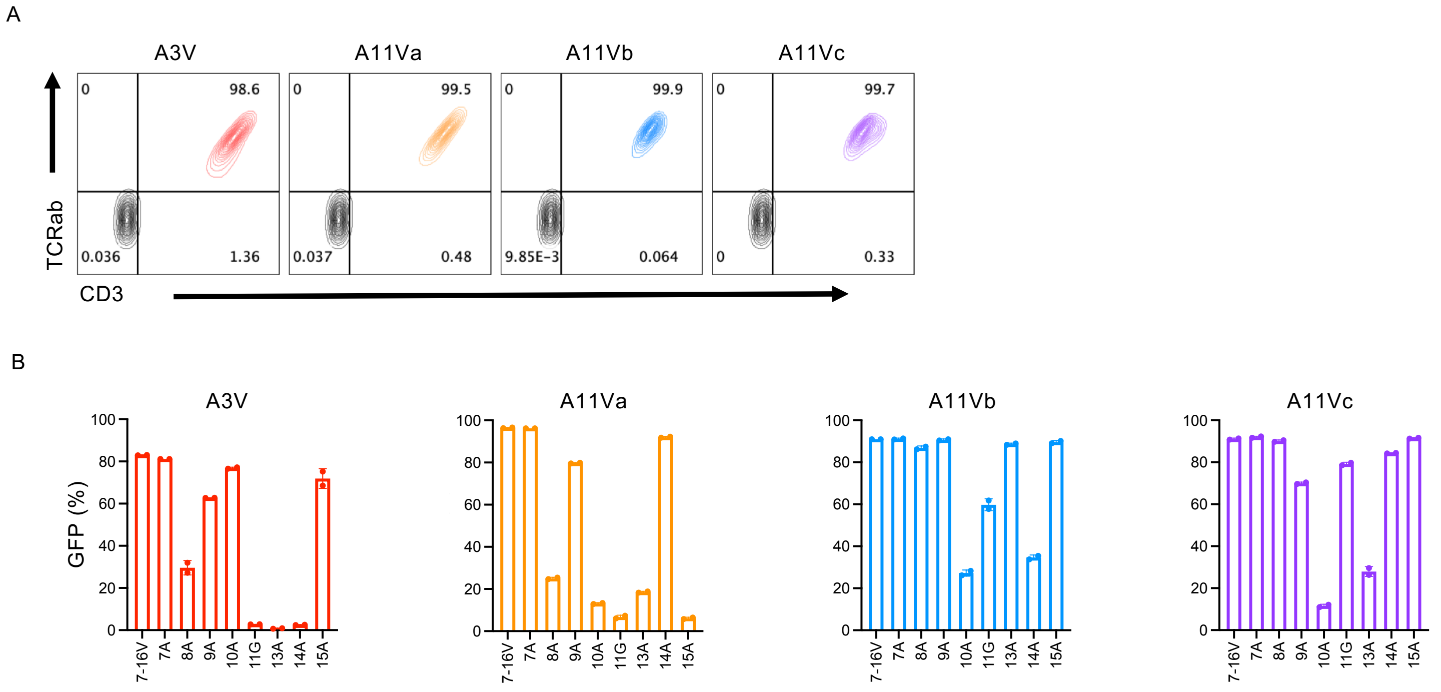

Supplemental Figure 2: Interrogation of TCR peptide binding motifs using J^ASP90_CD8+ cells.

(A) FACS profiles demonstrating CD3 and TCR β of TCR-engineered (colored) vs TCR^null J^ASP90_CD8+ following cell sorting.
(B) Bar graphs representing NFAT Activation as %GFP positive JASP90_CD8+ cells following 16h coculture with HLA-I matched K562 cells pulsed with Alanine / Glycine scanning library peptides.

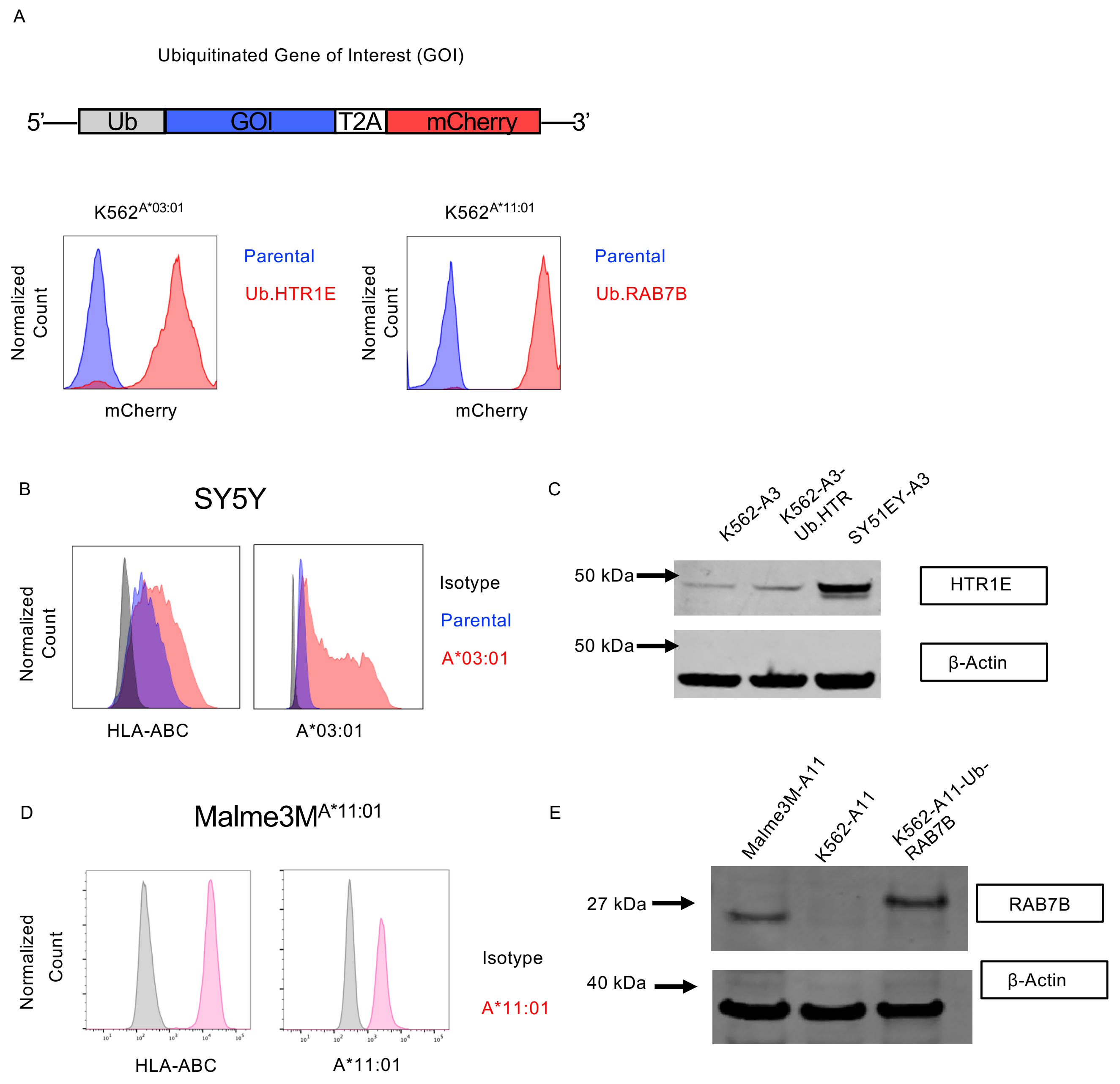

Supplemental Figure 3: Assessment of TCR cross-reactivity to non-cognate peptides.

(A) Schematic of lentiviral construct used to overexpress ubiquitinated HTR1E (Ub.HTR1E) and RAB7B (Ub.RAB7B) constructs. FACS plots demonstrating mCherry reporter expression in mono-allelic K562 cell lines following lentiviral transduction and cell sorting (Red) compared to non-transduced controls (Blue)
(B) FACS plots demonstrating HLA-ABC (W6/32) and HLA-A*03:01 expression by SY5Y cells engineered with lentiviral particles encoding HLA-A*03:01 SCD construct (Red) compared to non-transduced (Blue) and isoptype (Black) controls.
(C) Western blot demonstrating HTR1E expression in K562 (engineered) and SY5Y (endogenous) cell lines.
(D) FACS plots demonstrating HLA-ABC (W6/32, Red) and HLA-A*11:01 (One Lambda, cat#BIH0084, Red) expression by Malme-3M cells and isoptype controls are shown in grey.
(E) Western blot demonstrating RAB7B expression in K562 (engineered) and Malme-3M (endogenous) cell lines.

A

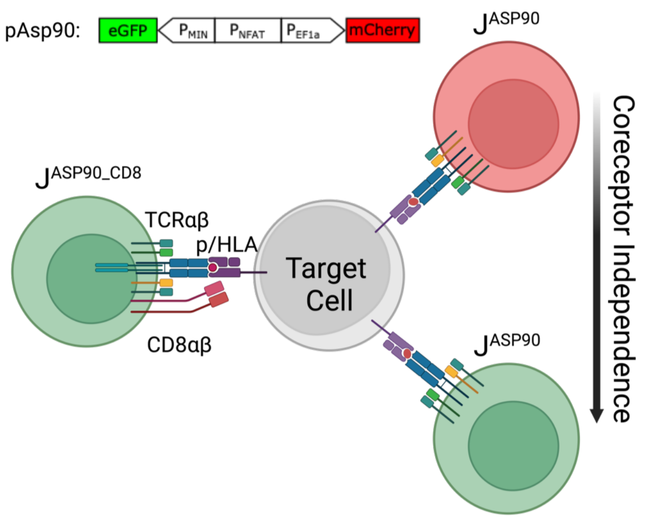

B

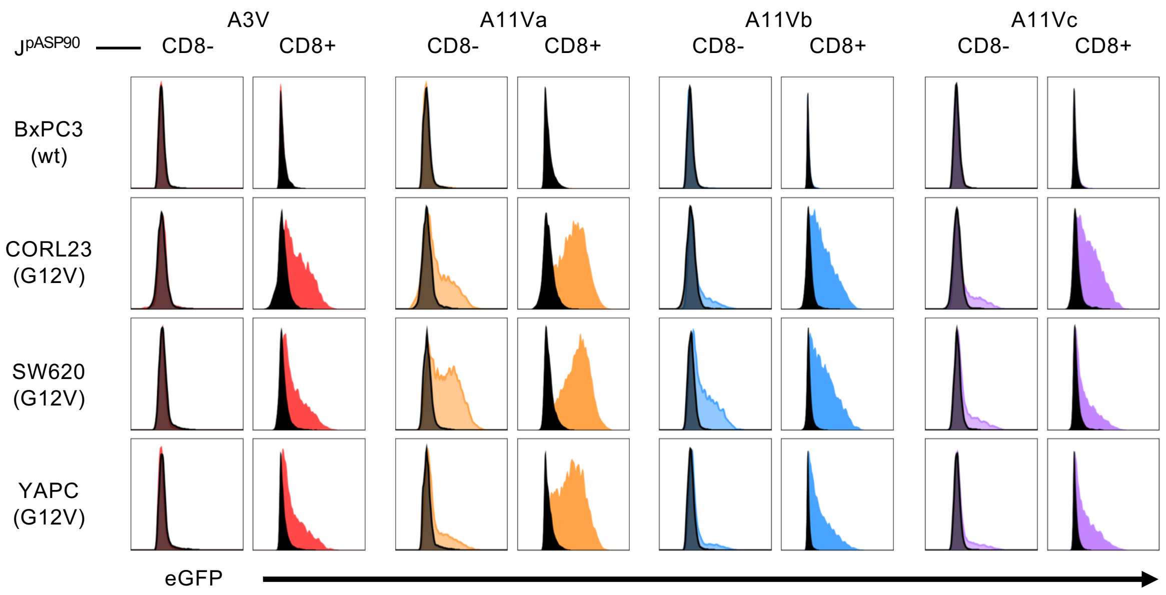

Supplemental Figure 4: Jurkat reporter system to evaluate TCR CD8 co-receptor independence.

(A) Schematic of Jurkat reporter system.
(B) FACS histogram plots demonstrating GFP expression of TCR-engineered J^{ASP90_CD8+} and J^{ASP90_CD8+} cells following co-culture with HLA-I matched (colored) vs unmatched (black) $KRAS^{WT}$ (BxPC3) or $KRAS^{G12V}$ (CORL23, SW620, YAPC) tumor cell lines.

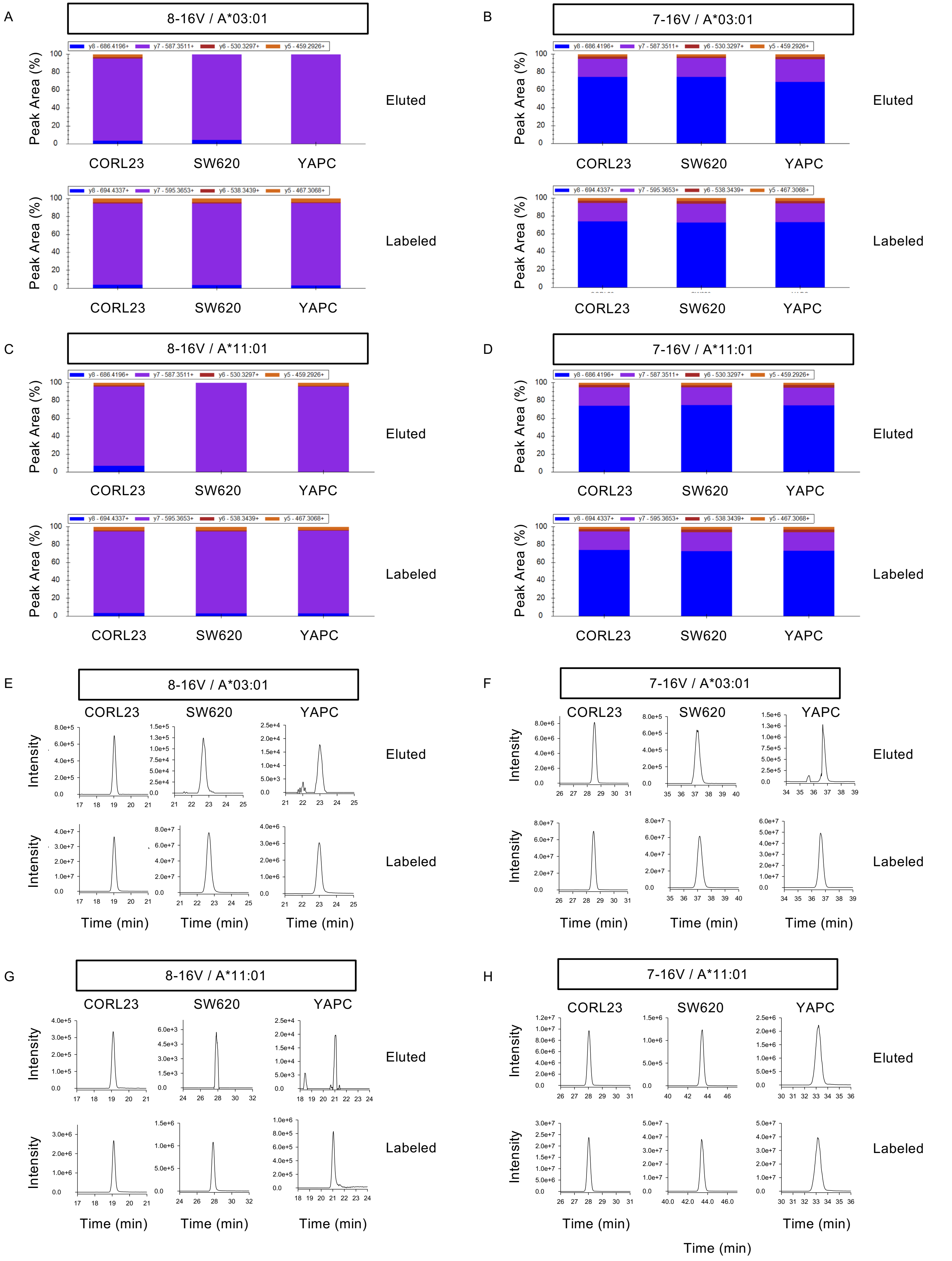
A
8-16V / A*03:01
B
7-16V / A*03:01
C
8-16V / A*11:01
D
7-16V / A*11:01
y8 - 686.4196+
y7 - 587.3511+
y6 - 530.3297+
y5 - 459.2926+
y8 - 694.4337+
y7 - 595.3653+
y6 - 538.3439+
y5 - 467.3068+
Peak Area (%)
CORL23
SW620
YAPC
Eluted
Labeled
E
8-16V / A*03:01
F
7-16V / A*03:01
G
8-16V / A*11:01
H
7-16V / A*11:01
Intensity
Time (min)

Supplemental Figure 5: Quantitation of 8-16V and 7-16V ions restricted to HLA-A*03:01 and HLA-A*11:01 eluted from KRASG12V tumor cell lines.

(A-D) Stacked ion fragment intensity plots of eluted vs stable labeled 8-16V or 7-16V ions from CORL23, SW620 and YAPC tumor cells expressing HLA-A*03:01 or HLA-A*11:01.
(E-H) Absolute quantification analysis of 8-16V and 7-16V ions eluted from CORL23, SW620 and YAPC tumor cells expressing HLA-A*03:01 or HLA-A*11:01. LC-PRM traces for the specific parent-to-product ion transitions for eluted 8-16V and 7-16V (Upper) along with internal standard peptides (Lower) are shown. Internal standard peptides (100 fmol) have a C-terminal stable labeled lysine (K^) of chemical composition $^{13}C_6{}^{15}N_2$.

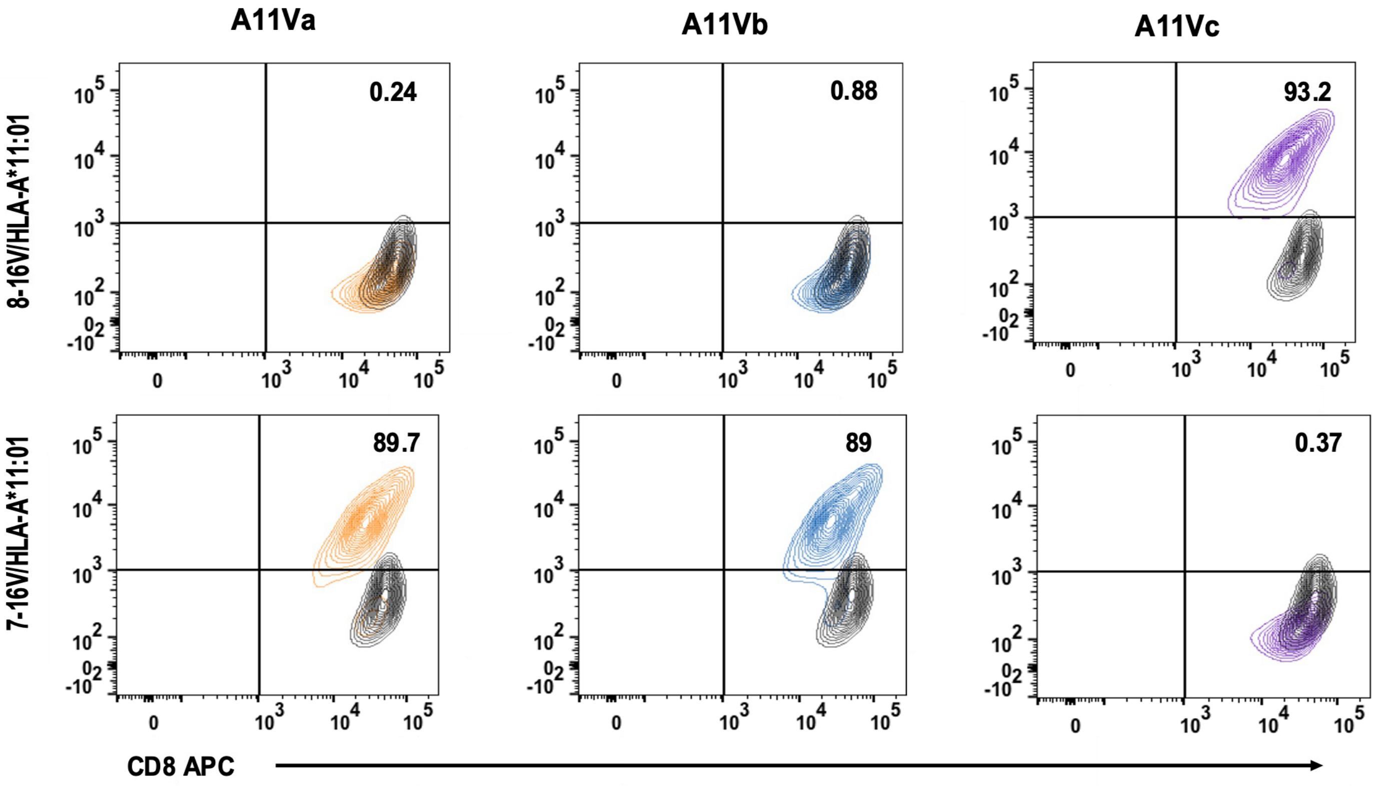

Supplemental Figure 6: CD8+ T cells redirected with A11Va-c TCRs differentially recognize nonamer or decamer KRAS^{MUT} peptides restricted to HLA-A*11:01.

FACS plots demonstrating pHLA multimer staining by TCR-engineered (colored) versus TCR^{null} (black) CD8+ T cells using nonamer 8-16V/A*11:01 multimer (top) or decamer 7-16V/A*11:01 multimers (bottom).

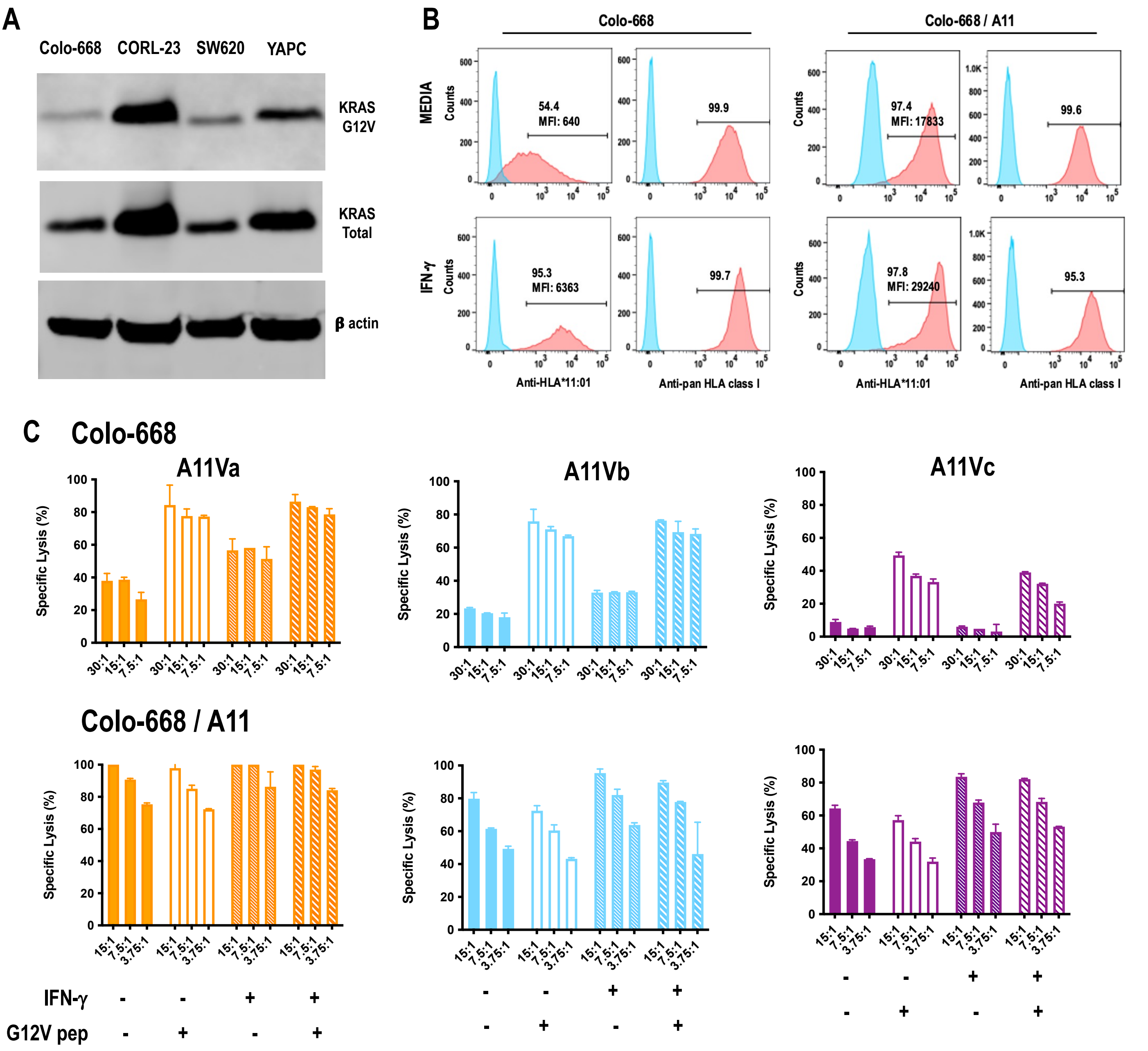
A
Colo-668
CORL-23
SW620
YAPC
KRAS G12V
KRAS Total
β actin
B
Colo-668
Colo-668 / A11
MEDIA
IFN-γ
Counts
54.4
MFI: 640
99.9
97.4
MFI: 17833
99.6
95.3
MFI: 6363
99.7
97.8
MFI: 29240
95.3
Anti-HLA*11:01
Anti-pan HLA class I
C
Colo-668
A11Va
A11Vb
A11Vc
Specific Lysis (%)
Colo-668 / A11
IFN-γ
G12V pep

Supplemental Figure 7: CD8+ T cells redirected with A11Va-c TCRs exhibit cytotoxic activity against Colo-668 cells which the naturally express KRASG12V and HLA-A*11:01.

(A) Total and G12V KRAS protein levels in COR-L23, Sw620, YAPC, and Colo-668 as determined by whole cell lysate in western blot assay. Each lane represents a total protein loading of 40ug as determined by Bradford assay. Beta-actin serves as control for protein loading.
(B) FACS plots demonstrating HLA-A*11:01 (clone: BIH0084) and pan-HLA class I (clone: W6/32) expression by Colo-668 and a HLA-A*11:01-engineered Colo-668 (Colo-668/A11) under steady-state (MEDIA) or upon IFN-g (24h, 100U/mL) treatment. Percentage of cells reactive with antibody as well as mean fluorescence index of HLA-A*11:01+ cancer cells is indicated.
(C) Cytotoxic activity as determined in a 4h 51-Cr-release assay of A11Va-c -engineered CD8+ T cells against Colo-668 (top) and Colo-668 / A11 (bottom) in steady state or upon IFN-g in the presence or absence of KRAS G12V peptide.

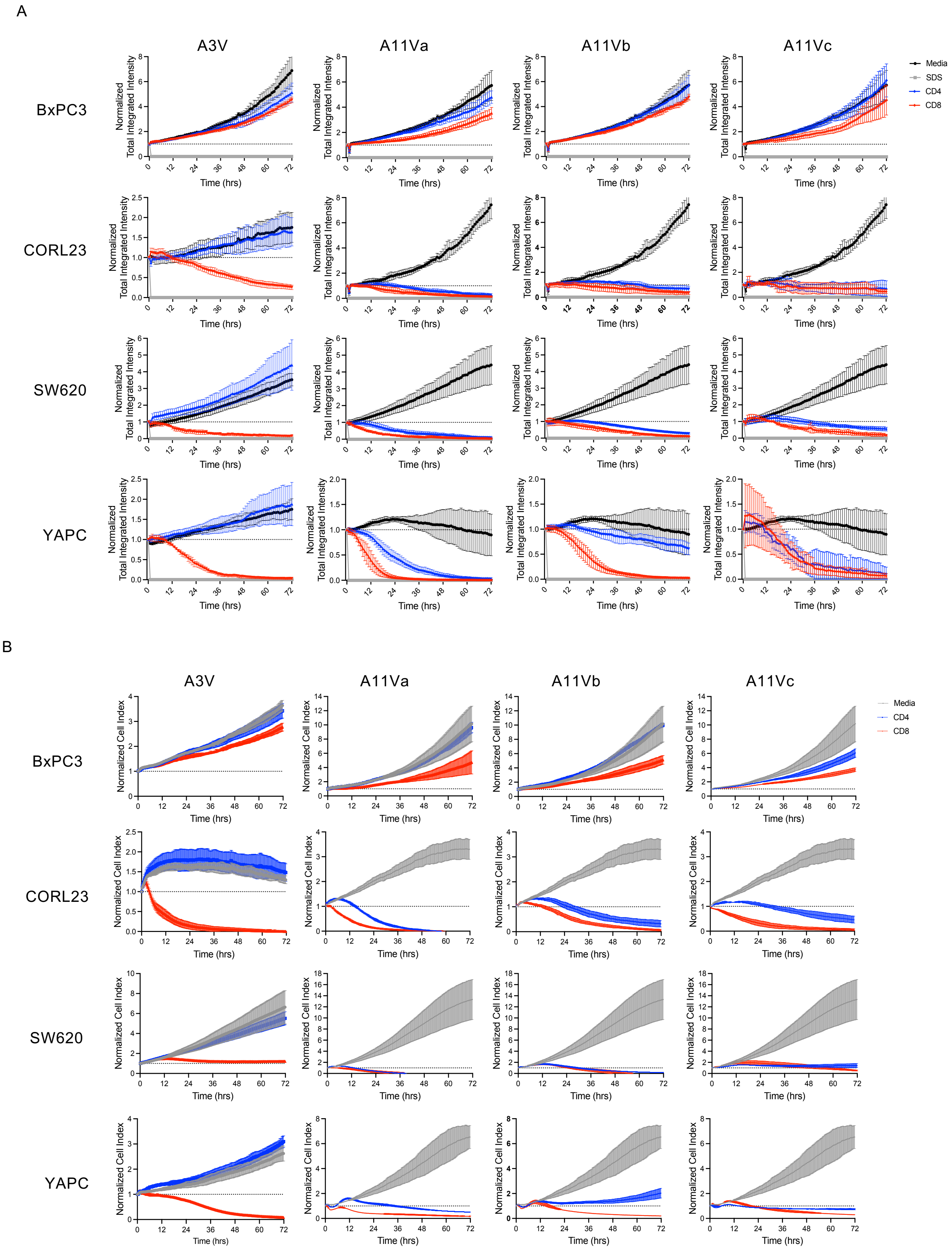

A
A3V
A11Va
A11Vb
A11Vc
Media
SDS
CD4
CD8
BxPC3
CORL23
SW620
YAPC
Normalized Total Integrated Intensity
Time (hrs)
B
Normalized Cell Index

Supplemental Figure 8: TCR redirected CD8+ and CD4+ T cells exhibit cytotoxic activity against KRAS G12V + tumor cell lines.

Real-time cell analysis over time following coculture of TCR-engineered CD8+ or CD4+ T cells with HLA-I matched BxPC3 (wt), CORL23 (G12V), SW620 (G12V) or YAPC (G12V) over a 72h period at a 3:1 effector to target ratio.
(A) Change in Normalized Green Total Integrated Intensity.
(B) Change in Normalized Cellular Impedance.

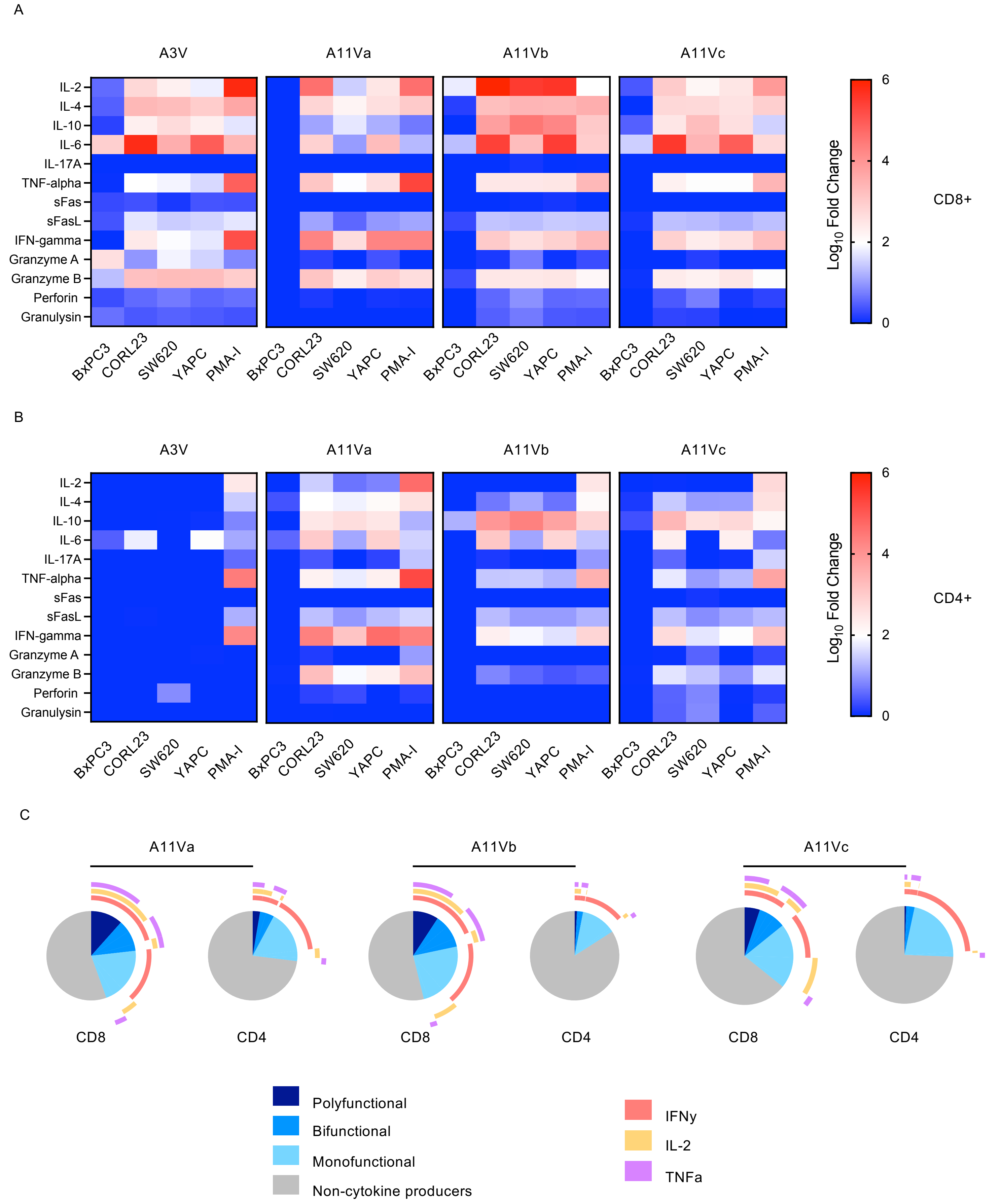

A
A3V
A11Va
A11Vb
A11Vc
IL-2
IL-4
IL-10
IL-6
IL-17A
TNF-alpha
sFas
sFasL
IFN-gamma
Granzyme A
Granzyme B
Perforin
Granulysin
BxPC3
CORL23
SW620
YAPC
PMA-I
Log_{10} Fold Change
6
4
2
0
CD8+
B
CD4+
C
CD8
CD4
Polyfunctional
Bifunctional
Monofunctional
Non-cytokine producers
IFNy
IL-2
TNFa

Supplemental Figure 9: Antigen-specific cytokine expression by TCR-redirected CD8+ and CD4+ T cells.

Cytokine bead array demonstrating cytokine expression by TCR-engineered (A) CD8+ or (B) CD4+ T cells following coculture with HLA-I matched BxPC3, CORL23, SW620, or YAPC cell lines. PMA-I is used as a positive control. Data is presented as Log10 Fold Change as compared to coculture with corresponding HLA-I mismatched cell lines.

(C) Intracellular cytokine staining of TCR-engineered CD8+ and CD4+ T cells following coculture with HLA-I matched CORL23 tumor cells. Pie charts represent the % CD8+ or CD4+ cytokine producing T cells as indicated within the figure legend.

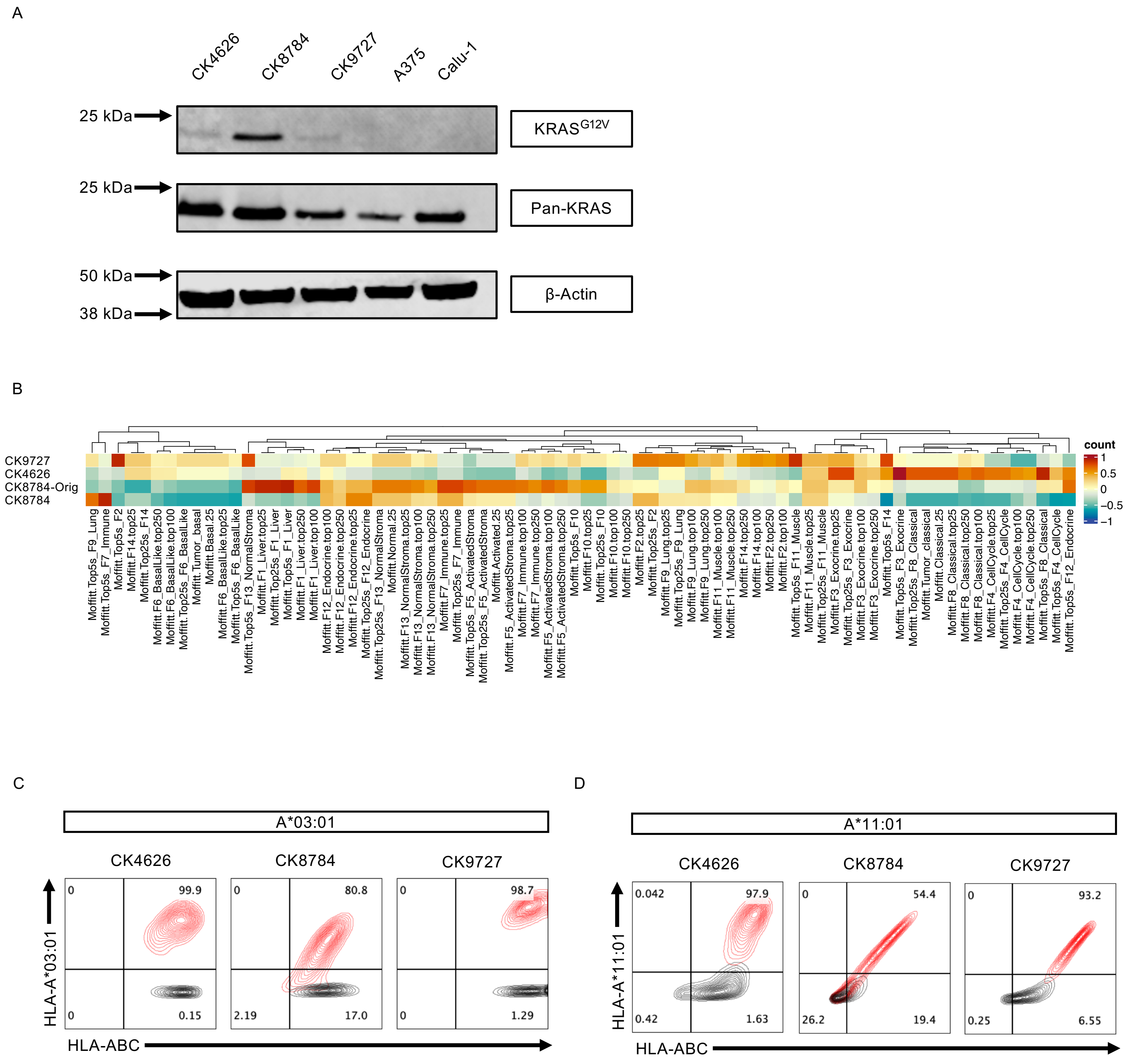

Supplemental Figure 10: Characterization of PAAD patient-derived cell lines.

(A) Western blot demonstrating $KRAS^{G12V}$ expression in PAAD PDCs. A375 (KRASWT) and Calu-1 (KRASG12C) serve as negative controls.

(B) Consensus clustered heat map of PDCs using correlation as the underlying distance function.

Surface expression of pan- and allele-specific HLA-I expression by (C) HLA-A*03:01 and (D) HLA-A*11:01 engineered PDCs (red) as compared to parental line (black) by FACS.

Characteristic	
Age at consent (years)	
Median (range)	63 (43-76)
Mean (SD)	63.03 (10.2)
Sex, n (%)	
Male	7 (78)
Female	2 (22)
Race/ethnicity, n (%)	
White	8 (89)
Black	1 (11)
Asian	0 (0)
Unknown	0 (0)
Anti-cancer therapy, n (%)	
Systemic therapy	9 (100)
Surgical Resection	9 (100)
Radiation therapy	5 (56)
Surgical procedure, n (%)	
Pancreaticoduodenectomy	6 (67)
Distal pancreatectomy w/ splenectomy	2 (22)
Total pancreatectomy w/ splenectomy	1 (11)
Pathology, n (%)	
Stage I	3 (33)
Stage II	3 (33)
Stage III	3 (33)
Lymph node status, n (%)	
N0	4 (44)
N1/2	5 (55)
Tumor KRAS mutation, n (%)	
G12D	3 (33)
G12V	4 (44)
G12R	1 (11)
G12C	1 (11)
HLA-I Enrollment Criteria, n (%)	
A*02:01	5 (56)
A*03:01	2 (22)
A*11:01	1 (11)
B*07:02	1 (11)
C*08:02	3 (33)

Supplemental Table 1: UPCC# 04218 subject demographics and clinical characteristics.
Demographic information reported as defined by investigator and specified by participant

Subject	$KRAS^{MUT}$	HLA-A	HLA-B	HLA-C	HLA-DR	HLA-DRw	HLA-DQB	HLA-DQA	HLA-DPA	HLA-DPB
2	G12V	03:01	07:02	04:01	01:01		05:01	01:01		04:01
		11:01	35:01	07:02	13:01	B3*01:01	06:03	01:03	01:03	20:01
		23:01	14:02	04:01	01:02		03:01	01:01		04:02
4	G12D	33:01	44:03	08:02	11:04		05:01	05:05	01:03	104:01
		02:01	13:02	01:02	01:03		02:02	02:01		04:01
6	G12D	30:56	27:05	06:02	07:01	B4*01:03	03:01	05:05	01:03	04:02
8	G12C	01:01	15:01	03:04	04:01	B4*01:03	03:02	01:03		
		02:01	32:01	12:02	15:02	B5*01:02	06:01	03:01	01:03	04:01
		33:01	14:02	08:02	01:02		03:01	01:01		02:01
12	G12V	33:03	18:01	12:03	11:04	B3*02:02	05:01	05:05	01:03	104:01
			40:01	03:04	04:01	B3*03:01	03:01	01:02		
13	G12D	02:01	44:02	05:01	13:02	B4*01:03	06:04	03:03	01:03	04:01
		02:01	15:01	03:03	04:01	B4*01:03	03:01	02:01	01:03	04:01
15	G12V	32:01	57:01	06:02	07:01	B4*01:03N	03:03	03:03	02:01	13:01
		23:01	14:02	08:02	01:02		03:01	01:01	01:03	04:01
21	G12R	26:01	41:02	17:03	11:04	B3*02:02	05:01	05:05	02:01	13:01
		02:01	14:01	04:01	07:01	B3*01:01	02:02			
24	G12V	03:01	25:08	07:01	13:01	B4*01:03	06:03			14:01

Supplemental Table 2: Subject tumor KRASMUT and HLA typing.

Subject	Vaccine Peptide								
	Number	ID	Protein	Sequence	Position	Length	Targeted HLA	Comments	Immune Response
2	1	gp17-25	gp100	ALLAVGATK	17-25	9	A*03:01	Control	Y
	2	NY60-72	NY-ESO-1	APRGPHGGAASGL	60-72	13	B*07:02	Control	Y
	3	8-16V	$KRAS^{G12V}$	VVGAVGVGK	8-16	9	A*03:01 / A*11:01		Y
	4	7-16V	$KRAS^{G12V}$	VVVGAVGVGK	7-16	10	A*03:01 / A*11:01		Y
4	1	10-18D	$KRAS^{G12D}$	GADGVGKSA	10-18	9	C*08:02		N
6	1	5-14D	$KRAS^{G12D}$	KLVVVGADGV	5-14	10	A*02:01		N
8	1	gp280-288	gp100	YLEPGPVTV	280-288	9	A*02:01	Control	Y
	2	5-14C	$KRAS^{G12C}$	KLVVVGACGV	5-14	10	A*02:01		N
	3	5-14D	$KRAS^{G12D}$	KLVVVGADGV	5-14	10	A*02:01		N
	4	5-14R	$KRAS^{G12R}$	KLVVVGARGV	5-14	10	A*02:01		N
	5	5-14V	$KRAS^{G12V}$	KLVVVGAVGV	5-14	10	A*02:01		N
12	1	10-18D	$KRAS^{G12D}$	GADGVGKSA	10-18	9	C*08:02		N
	2	10-19D	$KRAS^{G12D}$	GADGVGKSAL	10-19	10	C*08:02		N
	3	3-12R	$KRAS^{G12R}$	EYKLVVVGAR	3-12	10	A*33:01 / A*33:03		Y
	4	1-25D	$KRAS^{G12D}$	MTEYKLVVVGADGVGKSALTIQLIQ	1-25	25	HLA-II		Y
	5	1-25R	$KRAS^{G12R}$	MTEYKLVVVGARGVGKSALTIQLIQ	1-25	25	HLA-II		Y
	6	5-21V	$KRAS^{G12V}$	KLVVVGAVGVGKSALTI	5-21	17	HLA-II		Y
13	1	5-14D	$KRAS^{G12D}$	KLVVVGADGV	5-14	10	A*02:01		N
	2	5-14V	$KRAS^{G12V}$	KLVVVGAVGV	5-14	10	A*02:01		N
	3	1-25D	$KRAS^{G12D}$	MTEYKLVVVGADGVGKSALTIQLIQ	1-25	25	HLA-II		N
	4	1-25R	$KRAS^{G12R}$	MTEYKLVVVGARGVGKSALTIQLIQ	1-25	25	HLA-II		Y
15	1	5-14V	$KRAS^{G12V}$	KLVVVGAVGV	5-14	10	A*02:01		N
	2	5-6/8-14V	$KRAS^{G12V}$	KL_VVGAVGV	5-6/8-14	9	A*02:01	Spliced	N
	3	10-19D	$KRAS^{G12D}$	GADGVGKSAL	10-19	10	C*03:03		N
	4	10-19R	$KRAS^{G12D}$	GADGVGKSAL	10-19	10	C*03:03		N
	5	1-25D	$KRAS^{G12D}$	MTEYKLVVVGADGVGKSALTIQLIQ	1-25	25	HLA-II		Y
	6	1-25R	$KRAS^{G12R}$	MTEYKLVVVGARGVGKSALTIQLIQ	1-25	25	HLA-II		Y
	7	5-21V	$KRAS^{G12V}$	KLVVVGAVGVGKSALTI	5-21	17	HLA-II		N
21	1	10-18D(18V)	$KRAS^{G12D}$	GADGVGKSV	10-18	9	C*08:02	Anchor Modified	N
	2	10-19D	$KRAS^{G12D}$	GADGVGKSAL	10-19	10	C*08:02		N
	3	1-25D	$KRAS^{G12D}$	MTEYKLVVVGADGVGKSALTIQLIQ	1-25	25	HLA-II		Y
	4	1-25R	$KRAS^{G12R}$	MTEYKLVVVGARGVGKSALTIQLIQ	1-25	25	HLA-II		Y
	5	5-21V	$KRAS^{G12V}$	KLVVVGAVGVGKSALTI	5-21	17	HLA-II		Y
24	1	gp17-25	gp100	ALLAVGATK	17-25	9	A*03:01	Control	N
	2	7-16C	$KRAS^{G12C}$	VVVGACGVGK	7-16	10	A*03:01		N
	3	7-16D	$KRAS^{G12D}$	VVVGADGVGK	7-16	10	A*03:01		N
	4	7-16R	$KRAS^{G12R}$	VVVGARGVGK	7-16	10	A*03:01		N
	5	7-16V	$KRAS^{G12V}$	VVVGAVGVGK	7-16	10	A*03:01		N
	6	8-16V	$KRAS^{G12V}$	VVGAVGVGK	8-16	9	A*03:01		N
	7	5-21V	$KRAS^{G12V}$	KLVVVGAVGVGKSALTI	5-21	17	HLA-II		Y

Supplemental Table 3: HLA-I and HLA-II restricted control and KRASMUT peptides including in subject vaccines. Bold indicates positive immune responses.

TCR ID	Non-Cognate Antigen Binding Motif
A3V	{DEFNPWY}-[VCL]-[V ACILQR]-{DIVW}-A-[VCI]-[VCIMT]-{EFKLPWY}-K
A11Va	{KRWY}-[VCDGINW]-{DGHN}-G-[ACISTV]-[VCIQT]-G-[V ACILMST]-G-[KCHNRSY]
A11Vb	{FHKLR}-{FGHMQRWY}-{DGR}-G-[ACS]-[VCNT]-[GACHQ]-[VCL]-[GACPS]-[KCDINRY]
A11Vc	x-{FGLNWY}-[V ACGIL T]-G-[ACGLS]-[V ACHILMNQT]-G-{DEGHKR Y}-{L}-[KCR Y]

Supplemental Table 4: TCR non-cognate peptide binding motifs.

Pattern syntax is indicated as follows: ‘x’ is used for a position where any amino acid is accepted. Accepted amino acids for a given position are listed between square brackets ‘[ ]’. Amino acids that are not accepted at a given position are listed between curly brackets ‘{ }’. Each position in a pattern is separated from the subsequent position by a ‘-’.

TCR ID	Epitope	Co-receptor	EC50 (M)	EC50 95% CI (M)
A3V	7-16V	CD8+	7.39E-09	5.033e-9 to 1.073e-8
		CD8-	1.97E-07	1.379e-7 to 2.850e-7
A11Va	7-16V	CD8+	6.09E-10	3.574e-10 to 1.013e-9
		CD8-	9.00E-09	5.600e-9 to 1.441e-8
A11Vb	7-16V	CD8+	2.58E-10	1.893e-10 to 3.543e-10
		CD8-	2.36E-09	1.710e-9 to 3.278e-9
A11Vc	8-16V	CD8+	7.56E-10	3.429e-10 to 1.585e-9
		CD8-	8.64E-10	5.915e-10 to 1.252e-9
	7-16V	CD8+	7.45E-09	2.491e-9 to 2.021e-8
		CD8-	9.16E-09	5.000e-9 to 1.647e-8

Supplemental Table 5: Functional TCR avidities in the presence or absence of the CD8 co-receptor.

EC_{50} values determined by linear regression analysis of data presented in Figure 4A and represented in Figure 4B.

		KRAS								HLA					
Sample Number	Model	Protein	DNA	Reads	Variant Allelic Fraction	TP53	SMAD4	CDKN2A	Others	A1	A2	B1	B2	C1	C2
CK8784	485368-065-R4-J2-PDC.tumor	p.G12V	c.35G>T	649	1	p.E298*			ARID1 p.E1387Rfs*94 BACH2 p.R651*	02:01	23:01	44:03	18:01	07:01	04:01
CK9727	561559-040-R-J1-PDC.tumor	p.G12V	c.35G>T	240	0.56	p.M237I	p.E390_C391ins*	p.L16Rfs*6		01:01	23:01	44:03	57:01	06:02	04:01
CK4626	K24384-001-R-PDC.tumor	p.G12V	c.35G>T	259	0.65	p.Y236C			PMS2 p.H479Q KMT2 p.R4549H ATM p.S49C	24:02	24:02	07:02	07:02	07:02	07:02

Supplemental Table 6: Characterization of PAAD patient-derived cell lines using whole-exome sequencing data.
