# Supplementary material for: Natural TCRs targeting KRASG12V display fine specificity and sensitivity to human solid tumors
Source: J Clin Invest. 2024 Sep 17;134(21):e175790. doi: 10.1172/JCI175790 (PMC11529987; doi:10.1172/JCI175790)
Supplement: Unedited blot and gel images [file jci-134-175790-s012.pdf]

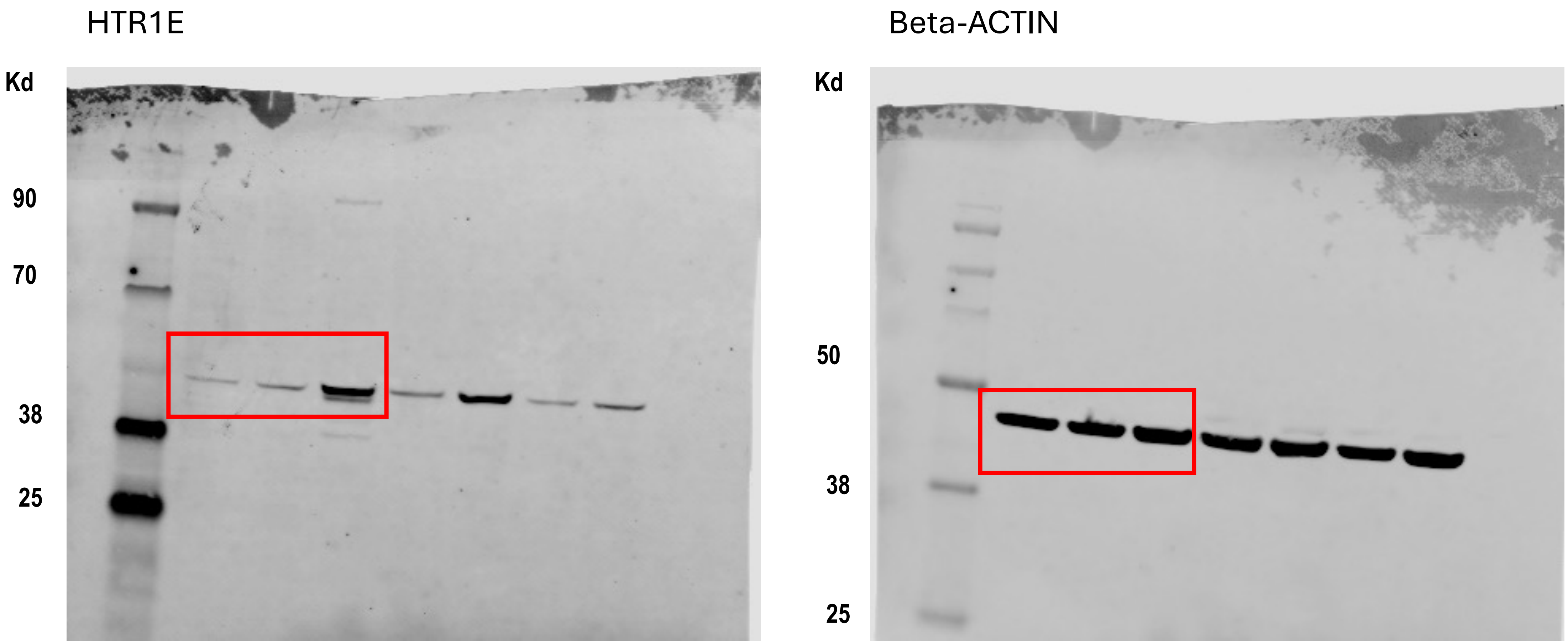

Supplementary Figure 3C

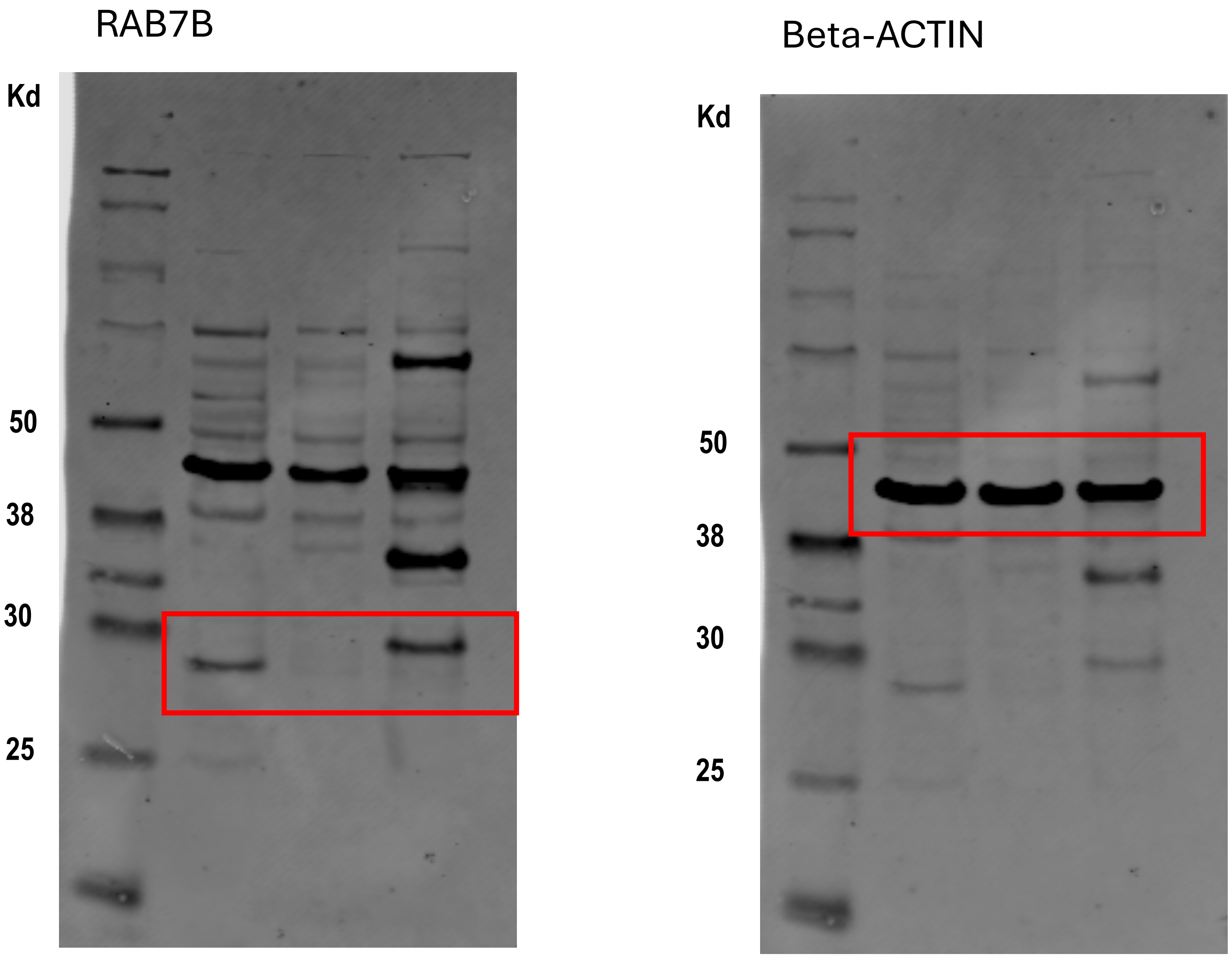

Supplementary Figure 3E

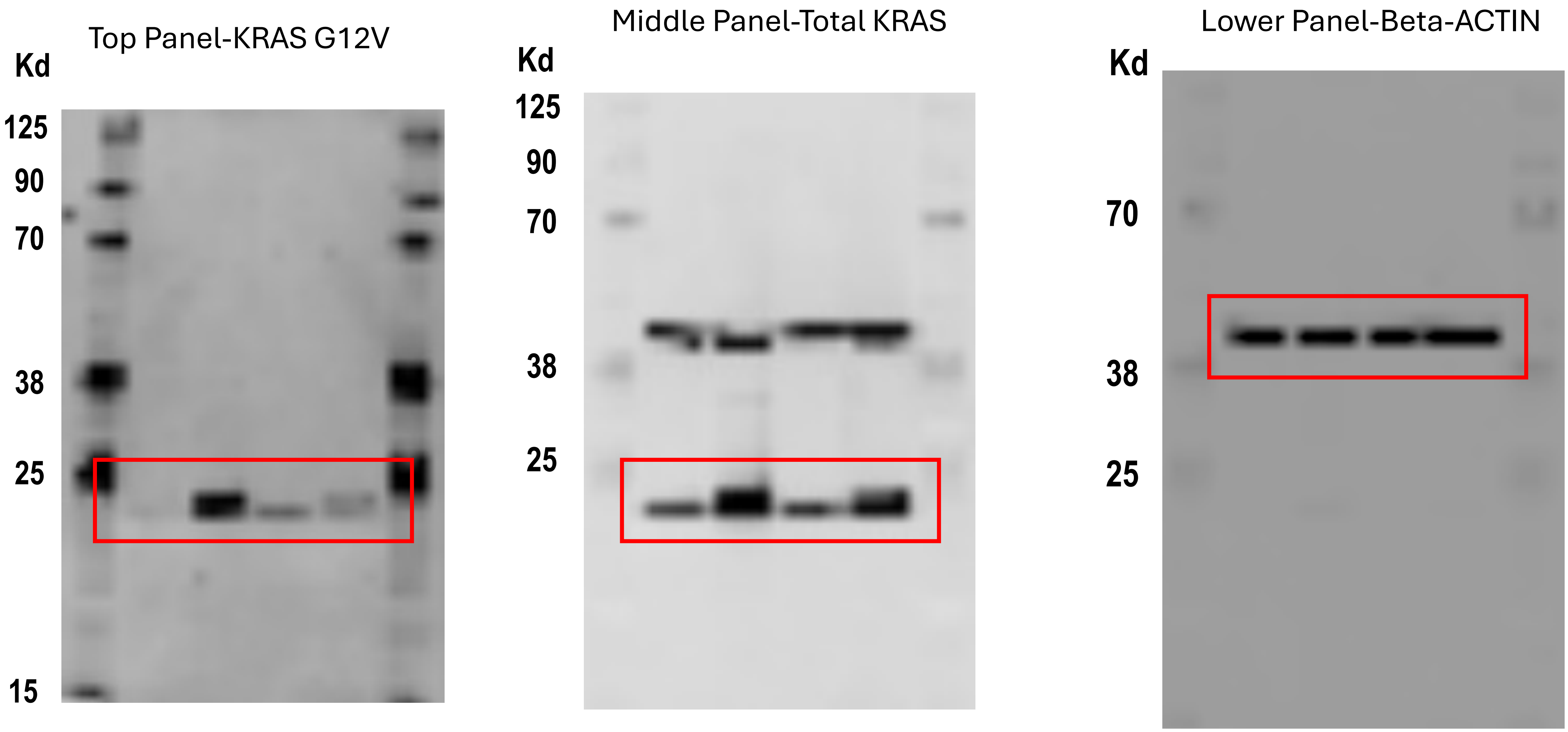

Supplementary Figure 7A

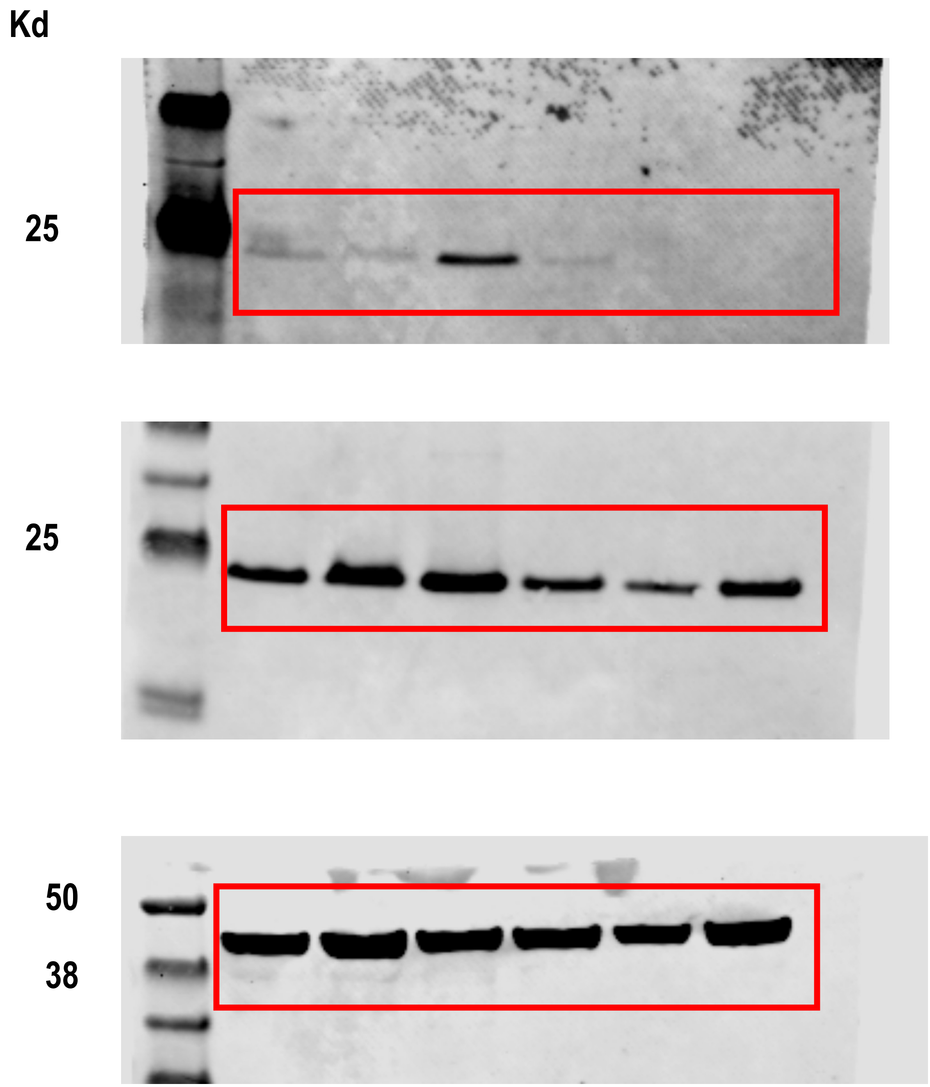

Supplementary Figure 10A
